# Maresin-1 promotes neuroprotection and modulates metabolic and inflammatory responses in disease-associated cell types in preclinical models of multiple sclerosis

**DOI:** 10.1016/j.jbc.2025.108226

**Published:** 2025-01-27

**Authors:** Insha Zahoor, Mohammad Nematullah, Mohammad Ejaz Ahmed, Mena Fatma, Mir Sajad, Kameshwar Ayasolla, Mirela Cerghet, Suresh Palaniyandi, Veronica Ceci, Giulia Carrera, Fabio Buttari, Diego Centonze, Yang Mao-Draayer, Ramandeep Rattan, Valerio Chiurchiù, Shailendra Giri

**Affiliations:** 1 Department of Neurology, Henry Ford Health, Detroit, Michigan, USA; 2 Division of Hypertension and Vascular Research, Department of Internal Medicine, Henry Ford Health, Detroit, Michigan, USA; 3 Department of Physiology, Wayne State University, Detroit, Michigan, USA; 4 Institute of Translational Pharmacology, National Research Council, Rome, Italy; 5 Laboratory of Resolution of Neuroinflammation, IRCCS Santa Lucia Foundation, Rome, Italy; 6 Department of Systems Medicine, University of Rome Tor Vergata, Rome, Italy; 7 Unit of Neurology, IRCCS Neuromed, Pozzilli (Is), Italy; 8 Oklahoma Medical Research Foundation, Oklahoma, Farmington Hills, Michigan, USA; 9 Women’s Health Services, Henry Ford Health, Detroit, Michigan, USA

**Keywords:** DHA, EAE, IL-10, inflammation, Maresin1, MS, resolution, therapeutics, Metabolism, SCENITH, SPM

## Abstract

Multiple sclerosis (MS) is a prevalent inflammatory neurodegenerative disease in young people, causing neurological abnormalities and impairment. To investigate a novel therapeutic agent for MS, we observed the impact of maresin 1 (MaR1) on disease progression in a well-known, relapsing-remitting experimental autoimmune encephalomyelitis mouse model. Treatment with MaR1 accelerated inflammation resolution, reduced neurological impairment, and delayed disease development by reducing immune cell infiltration (CD4+IL-17+ and CD4+IFNγ+) into the central nervous system. Furthermore, MaR1 administration enhanced IL-10 production, primarily in macrophages and CD4+ cells. However, neutralizing IL-10 with an anti-IL-10 antibody eliminated the protective impact by MaR1 in relapsing-remitting experimental autoimmune encephalomyelitis model, implying the significance of IL-10 in MaR1 treatment. Metabolism has been recognized as a critical mediator of effector activity in many types of immune cells. In our investigation, MaR1 administration significantly repaired metabolic dysregulation in CD4+ cells, macrophages, and microglia in EAE mice. Furthermore, MaR1 treatment restored defective efferocytosis in treated macrophages and microglia. MaR1 also preserved myelin in EAE mice and regulated O4+ oligodendrocyte metabolism by reversing metabolic dysregulation *via* increased mitochondrial activity and decreased glycolysis. Overall, in a preclinical MS animal model, MaR1 therapy has anti-inflammatory and neuroprotective properties. It also induced metabolic reprogramming in disease-associated cell types, increased efferocytosis, and maintained myelination. Moreover, our data on patient-derived peripheral blood mononuclear cells substantiated the protective role of MaR1, expanding the therapeutic spectrum of specialized proresolving lipid mediators. Altogether, these findings suggest the potential of MaR1 as a novel therapeutic agent for MS and other autoimmune diseases.

Multiple sclerosis (MS) is one of the most common inflammatory and neurodegenerative diseases in young adults and leads to the development of neurological defects accompanied by irreversible disability (1, 2). Although the relapsing–remitting phenotype of MS is treatable, the disease remains largely incurable, and progressive disability is inevitable. Patients commonly suffer from relapses accompanied by damaging inflammation, and current immunomodulatory medications suppress the entire immune system, resulting in severe side effects with subpar tolerability (3). Inflammation plays a detrimental role in several autoimmune diseases, including MS, necessitating investigations of endogenous inflammatory pathways (4). Over the course of disease, the body is pushed into remission, or into chronic disease state, by an imbalance between inflammation and endogenous defense mechanisms. The endogenous mechanisms that combat inflammation in MS are not sufficient to resolve this disease, resulting in chronic inflammation. Unresolved inflammation is the pathological hallmark of MS and several other autoimmune diseases; however, current therapeutic options fail to adequately suppress ongoing inflammation, resulting in inflammatory attacks that gradually increase in severity, causing continuous neuronal damage and preventing adequate remyelination, consequently leaving axons demyelinated and vulnerable to degeneration (5, 6). Therefore, investigating pathways that reduce excessive inflammation and promote rapid resolution is necessary.

Inflammation resolution is an active process mediated by endogenously derived metabolites of dietary polyunsaturated omega fatty acids (PUFAs), collectively termed specialized proresolving lipid mediators (SPMs). Dietary factors are modifiable environmental contributors to MS (7, 8). A large prospective study revealed a significant inverse association between PUFA intake and the risk of MS (9), suggesting that low PUFA intake may be a modifiable risk factor for MS. In-depth research on dietary factors is critical for determining the specific mechanisms of disease etiopathogenesis. Compelling evidence suggests defects in docosahexaenoic acid (DHA) metabolism, resulting in downstream SPM (resolvins, protectins, and maresins) deficiency and leading to chronic inflammation and delayed healing/repair processes (10, 11). However, information regarding the presence or abundance of these metabolites in patients with MS is limited (12).

We previously demonstrated that the biosynthesis of the DHA-derived metabolite and pathway marker for SPM maresins (MaRs), 14-HDoHE, is significantly reduced in patients with relapsing-remitting MS (RR-MS) (13). It is also reported that plasma maresin 1 (MaR1) levels were undetectable in patients with either RR-MS or progressive MS (14). MaR1 mitigates inflammatory damage in other disease models, with promising neurological recovery. This is achieved by promoting resolution and neuroprotection, as well as attenuating neuroinflammation and neurocognitive dysfunction (15, 16, 17). As a family of anti-inflammatory and proresolving lipid mediators, MaRs are a class of 14S-dihydroxyl–containing molecules with conjugated triene double bonds synthesized from DHA through an oxidative (*e.g.*, lipoxygenase-related) pathway during inflammation regression. They are highly conserved resolution mediators with potent anti-inflammatory and proresolving properties that contribute to tissue regeneration in acute or chronic inflammatory–related disease models (18, 19, 20). Therefore, administering MaR1, a potent self-limiting inflammatory factor, may be highly promising as an anti-inflammatory intervention strategy. In brief, MaR1 is synthesized from DHA by 12-LOX, with 14-HpDHA serving as its precursor and 14-HDHA serving as a byproduct (pathway marker). It binds to the receptor leucine-rich repeat-containing G protein-coupled receptor 6 (LGR6), and further studies are necessary to elucidate whether the 12-LOX–14-HDHA–LGR6 pathway is a crucial metabolic signaling axis that contributes to MaR1 bioaction regulation in peripheral inflammation and neuroinflammation. If so, this may provide an attractive therapeutic target(s) with potential clinical applications for MS treatment.

This study aimed to establish a comprehensive protective mechanism for MaR1 in a relapsing-remitting (RR) animal model of experimental autoimmune encephalomyelitis (EAE) and in peripheral blood mononuclear cells (PBMCs) obtained from RR-MS patients.

## Results

### MaR1 ameliorates clinical signs of EAE, improves pathophysiology, and promotes neuroprotection

**Figure 1:**
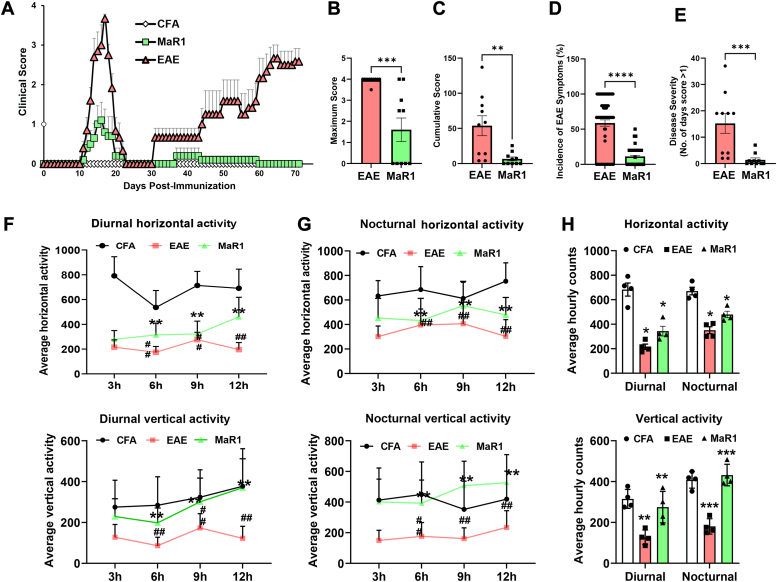
**Protective effect of MaR1 treatment on neurological deficits in the RR-EAE mouse model.***A*, clinical scores of SJL mice in the CFA, EAE, and EAE groups treated with MaR1 for 70 days after disease induction. EAE mice developed disease symptoms beginning on day 9 after immunization with PLP_139-151_, whereas MaR1-treated mice showed symptoms on day 10 (n = 10). *B*, maximum score. *C*, cumulative score. *D*, incidence of clinical symptoms. *E*, disease severity scores of EAE- and MaR1-treated mice. ∗∗*p* < 0.01, ∗∗∗*p* < 0.001, ∗∗∗∗*p* < 0.0001 (as determined by the Mann‒Whitney test) versus CFA and the MaR1 EAE. The data are shown as the mean ± SEM. For spontaneous motor activity, before the end of the experiment, mouse locomotor activity was measured during the day and nighttime to evaluate disease severity. *F*, average diurnal horizontal and vertical activities were measured in CFA-, EAE-, and MaR1-treated mice. *G*, average nocturnal horizontal and vertical activity was measured in CFA-, EAE-, and MaR1-treated mice. *H*, average hourly horizontal and vertical activity (diurnal and nocturnal) in CFA-, EAE-, and MaR1-treated mice. The values are expressed as the means ± SDs (N = 10). Statistical analyses were performed with one-way ANOVA and two-way ANOVA. ∗*p* < 0.05, ∗∗*p* < 0.01 *versus* the CFA group; #*p* < 0.05, ##*p* < 0.01 *versus* the MaR1-treated group.

To investigate the comprehensive protective mechanism of MaR1 in EAE, we induced EAE in SJL mice, a well-known relapsing-remitting animal model (RR-EAE) of MS. MaR1 treatment began 6 days after immunization, with a separate group of immunized mice receiving vehicle (0.1% ethanol). We found that MaR1-treated mice exhibited significant protection against neurological impairments (Fig. 1, *A*–*E*). EAE in SJL mice followed a classical RR pattern of disease (Fig. 1*A*). Compared with vehicle-treated EAE mice, MaR1 treatment did not affect early-onset disease; however, MaR1 treatment significantly reduced disease severity at the peak stage (Fig. 1*A*). MaR1 treatment effectively prevented further relapses in the treated group (Fig. 1*A*). Compared with EAE vehicle-treated mice, MaR1-treated mice had significantly lower cumulative scores (*p* < 0.01) and maximum scores (*p* < 0.001) (Fig. 1, *B* and *C*). Compared with vehicle treatment, MaR1 treatment significantly reduced disease severity (*p* < 0.001) and symptom incidence (*p* < 0.0001) in EAE mice (Fig. 1, *D* and *E*). Furthermore, we measured the effect of MaR1 on locomotor activity using the infrared-based automated activity monitoring system (21). The average horizontal and vertical activity during the day was significantly lower in EAE mice than in animals without EAE (injected with Complete Freund’s Adjuvant [CFA] alone) or those treated with MaR1. MaR1 treatment increased the horizontal and vertical activity of EAE mice (Fig. 1*F*). Compared with those of EAE mice, the nocturnal horizontal and vertical activities of EAE mice were significantly lower than those of non-EAE (CFA) mice; however, compared with EAE mice, MaR1-treated EAE mice showed a significant improvement in activity (Fig. 1*G*). We also measured hourly changes in horizontal and vertical activity during the day and night. We discovered a significant decrease in average hourly activities in untreated EAE mice compared with those in EAE mice, which was ameliorated by MaR1 treatment (Fig. 1*H*). Overall, MaR1 treatment improved neurological function in EAE mice and prevented disease progression in the RR-EAE animal model.

### MaR1 reduces the antigen-specific immune response and immunomodulates infiltrating cells in the CNS

**Figure 2:**
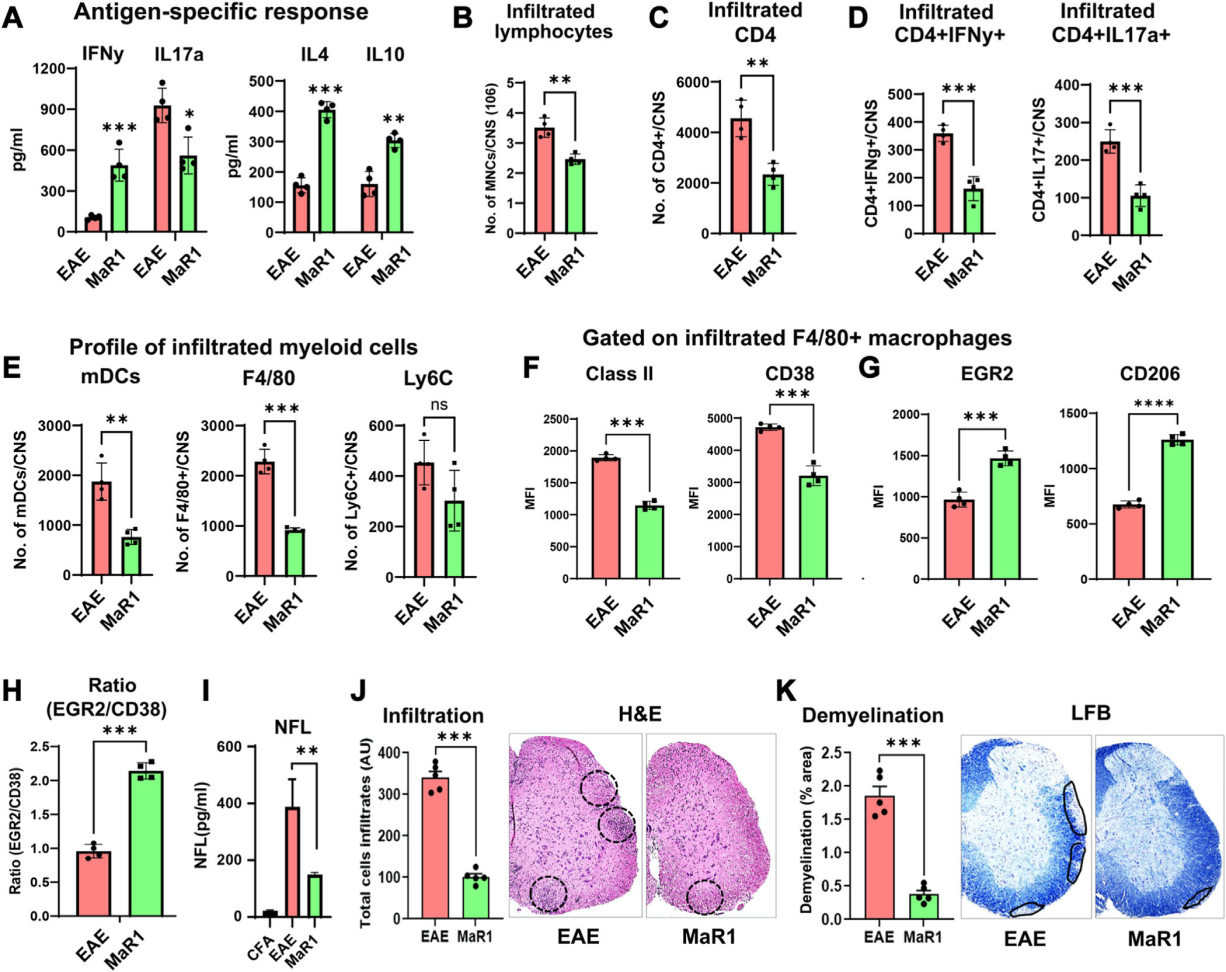
**MaR1 modulates antigen-specific responses and abrogates the infiltration of IL-17- and IFNγ-producing CD4+ T cells.***A*, the antigen recall response of spleen/LN cells was examined on day 17 after immunization for 72 h in the presence of PLP_139-151_ (n = 4). *B*–*D*, CNS tissues (brain and spinal cord) were processed, and the total number of leukocytes (CD45+) and CD4+ T cells and the frequencies of Th1 and Th17 cells in the CNS in treated and vehicle-treated RR-EAE mice (n = 4) were examined. *E*, infiltrating myeloid cells, including monocytic DCs, F4/80+ macrophages, and monocytes, were profiled in both treated and untreated EAE mice (N = 4). *F* and *G*, at the peak of the disease, the pro-inflammatory (class II and CD38) and anti-inflammatory (EGR2 and CD206) phenotypes of splenic macrophages (CD11b^+^F4/80^+^) were examined by flow cytometry, and the data are presented as a bar graph of the mean fluorescence intensity (MFI) (n = 4). *H*, the ratio of EGR2+/CD38+ macrophages was plotted to determine the macrophage phenotype (n = 4). *I*, the level of neurofilament-light chain (NFL) in the plasma of EAE model mice treated with or without MaR1 was examined *via* SIMOA (n = 5). *J* and *K*, spinal cord sections showing inflammatory infiltrates (H&E) and demyelination (LFB) (N = 5). Representative images showing histopathological changes in spinal cord tissue from EAE mice treated with MaR1 or vehicle. The circles indicate infiltration of inflammatory cells in (*J*) and demyelination of the nerves in (*K*). The percentage of demyelinated area was calculated as per previous publications. Scale bar represents 200 μm. The data are shown as the mean ± SEM. ∗*p* < 0.05, ∗∗*p* < 0.01, ∗∗∗*p* < 0.001, ∗∗∗∗*p* < 0.0001.

To investigate the effect of MaR1 on the myelin-specific immune response, we isolated splenic and lymph node (LN) cells from both the EAE- and MaR1-treated groups and stimulated them with PLP_139-151_. After 72 h of stimulation, the cell supernatant was tested for pro- and anti-inflammatory cytokines. MaR1 treatment decreased antigen-induced IL-17a production in spleen and LN cells while increasing the expression of IFNγ and anti-inflammatory cytokines, such as IL-4 and IL-10 (Figs. 2*A* and S3, *A* and *B*). This observation was further supported by quantitative PCR (qPCR) of pro- and anti-inflammatory cytokine levels in cells from the spleen and LNs (Fig. S3*C*). These findings indicate that MaR1 treatment affects antigen-specific immune responses.

Since T cells and myeloid cells contribute to autoimmune central nervous system (CNS) inflammation and demyelination (22), we used flow cytometry to analyze infiltrating cells in the CNS. We found that MaR1 treatment greatly reduced the infiltration of leukocytes (CD45+) into the CNS (Fig. 2*C*). Because CD4+ T cells play an important role in EAE disease pathogenesis, we measured the total number of infiltrating CD4+ T cells and their Th subsets, including Th1 and Th17 subsets, in CNS tissue from vehicle- and MaR1-treated EAE mice. MaR1 treatment significantly decreased the total number of infiltrating CD4+ (CD45 + CD4+) and IFNγ-expressing (Th1) and IL-17–expressing (Th17) CD4+ T cells in the CNS tissue of EAE model mice (Fig. 2, *C* and *D*), indicating that this proresolving mediator affects pathogenic CD4+ T cells. We also examined the effects of MaR1 treatment on myeloid cell infiltration and found that the numbers of monocytic dendritic cells (CD45 + CD11b + CD11c + MHC II+) and macrophages (CD45 + CD11b + F4/80+) were significantly reduced, but the number of infiltrated monocytes (CD45 + CD11b + Ly6G-Ly6C+) was not affected (Fig. 2*E*). Monocytes/macrophages are major effectors of demyelination in both MS and EAE and are highly plastic in nature (23, 24). Depending on the environment, monocytes differentiate into either pro-inflammatory or anti-inflammatory macrophages, and their ratio affects the outcome of the disease (25). Since anti-inflammatory macrophages ameliorate the clinical symptoms of EAE (25, 26), we examined the splenic nature of macrophages in the MaR1-treated and untreated EAE groups by investigating the expression of the pro-inflammatory markers MHC-II and CD38 (27) and the anti-inflammatory markers CD206 and EGR2 (27, 28). Notably, MaR1 treatment not only reduced MHC-II and CD38 expression and concomitantly induced EGR2 and CD206 expression (Fig. 2, *F* and *G*) but also increased the EGR2/CD38+ and CD206/CD38+ macrophage ratios in the treated group compared with those in the untreated EAE group (Figs. 2*H*, S3*D*), suggesting that MaR1 promotes a shift in macrophage polarization.

Blood neurofilament light chain (NFL) is a neuronal damage marker that has been linked to relapses, deterioration of the EDSS score, lesions on MRI images, and atrophy of both the brain and spinal cord in MS patients (29, 30, 31). Using very sensitive single-molecule array (SiMoA) technology, we found that MaR1 treatment significantly reduced NFL levels compared with those in the EAE group (Fig. 2*I*). The clinical score of MaR1-treated EAE mice was lower than that of vehicle-treated EAE mice, which was further supported by the decrease in the number of infiltrating leukocytes (Fig. 2*B*) and preservation of the myelin content in spinal cord sections, as observed through H&E and Luxol fast blue staining, respectively (Fig. 2, *J*–*L*). Histopathological analyses revealed that MaR1 treatment significantly reduced the number of immune cells in EAE mice compared with vehicle-treated EAE mice (Fig. 2*J*). Furthermore, the occurrence of demyelination was considerably lower in MaR1-treated mice than in vehicle-treated EAE mice (Fig. 2*L*). Overall, MaR1 effectively inhibited EAE disease progression by reducing the infiltration of immune cells into the CNS, blocking Th1 and Th17 immune responses, polarizing macrophages toward an anti-inflammatory phenotype, and providing protection against neuroaxonal injury and myelin loss.

### MaR1 shows therapeutic potential in the RR-EAE model by impeding the encephalitogenic property of CD4 effector cells

**Figure 3:**
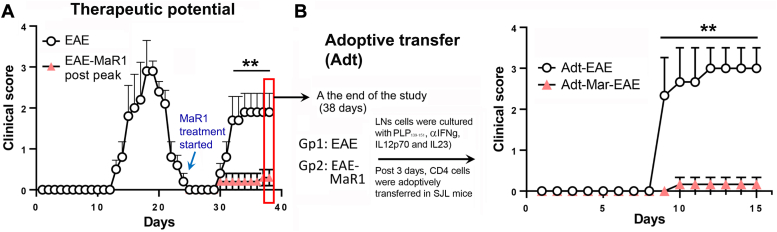
**MaR1 treatment impedes the encephalitogenic property of CD4 effector cells.***A*, EAE was induced in SJL mice, and MaR1 treatment began when the mice in the randomly divided groups had recovered from the disease on day 22 after immunization. *B*, at the end of the study (∼38 days), isolated LN cells from both groups were cultured with PLP_139-151_, anti-IFNy (10 μg/ml), IL-12p70, and IL-23 (10 ng/ml). After 3 days, the enriched CD4+ cells were injected into SJL mice (n = 5), after which the clinical score was monitored. Note: The Adt-EAE-MaR1 group was not treated with MaR1. ∗∗*p* < 0.01.

To investigate the therapeutic effect of MaR1 on EAE, we treated RR-EAE animals in remission on day 20 after immunization, when all the mice had recovered from paralysis, and the clinical score was monitored during subsequent relapse (30–38 dpi). We observed that MaR1 treatment significantly reduced relapse in RR-EAE mice, with animals showing a clinical score close to 0 (Fig. 3*A*). To better understand how MaR1 affects CD4 T-cell effector function, we isolated cells from LNs on day 38 and cultured them under Th17-inducing conditions with PLP_139-151_ (20 μg/ml) (Fig. 3*B*). After 3 days, CD4+ T cells were isolated and adoptively transferred into naïve SJL mice, and the clinical score was monitored daily. CD4+ T cells obtained from EAE mice were able to induce EAE, whereas those from the MaR1-treated group were incapable of inducing EAE (*p* < 0.01) (Fig. 3*B*). These findings indicate that MaR1 has therapeutic potential and that treatment affects the encephalitogenic properties of effector CD4+ T cells.

### MaR1 mediates its protection by producing IL-10 in EAE

**Figure 4:**
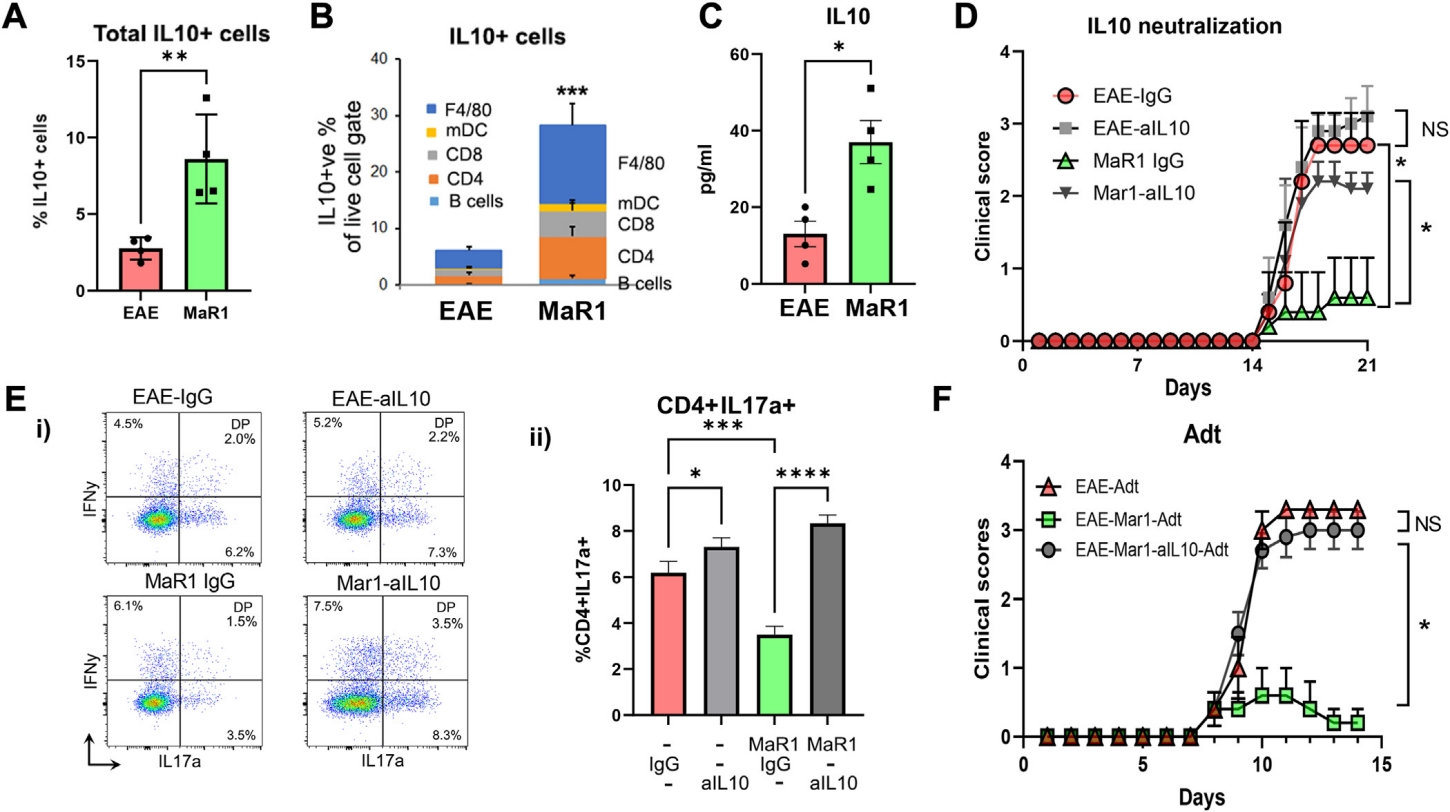
**Effect of IL-10 neutralization on the protective effect of MaR1 on EAE.***A* and *B*, spleen cells from EAE mice treated with or without MaR1 (day 18) were stimulated with PMA/ionomycin in the presence of GolgiPlug for 4 h and subjected to surface staining for CD4 (CD3+CD4+), CD8 (CD3+CD4+), B cells (CD3-CD19+), mDCs (CD11b + CD11c + Class II+) and macrophages (F4/80+), and intracellular staining for IL-10. CD45+IL-10+ cells were gated from live cells; on the basis of surface markers, all cell types expressing IL-10 were profiled (n = 4). *C*, plasma levels of IL-10 were measured *via* the SIMOA on day 70 in EAE mice treated with MaR1 or vehicle (n = 4). *D*, disease severity plot showing abrogation of the protective effect of MaR1 by an IL-10 neutralizing antibody compared with that of an IgG neutralizing antibody (N = 8). *E* i-ii, the number of CNS-infiltrating Th17 cells in all groups was determined *via* flow cytometry; the data are presented as a bar graph (n = 5). ∗*p* < 0.05 compared with IgG group; ∗∗∗∗*p* < 0.00001 compared with MaR1 IgG group. *F*, adoptive transfer of antigen-specific CD4+ T cells derived from the IL-10 neutralization experimental batch and monitoring of clinical scores (N = 5). ∗*p* < 0.05 versus vehicle EAE. The data are shown as the mean ± SEM. ∗∗*p* < 0.01, ∗∗∗*p* < 0.001 compared with the EAE group.

Because MaR1 promotes the expression and production of IL-10 (Figs. 2*A*, S3*C*), which can influence Th17 pathogenicity in autoimmune disorders (32), we next investigated the immune cells that produce IL-10 in response to MaR1 treatment. To do this, spleen cells from the vehicle- and MaR1-treated EAE groups were stimulated with PMA/ionomycin, followed by intracellular IL-10 staining and surface staining for various immune cells. MaR1 treatment dramatically increased the percentage of total IL-10–producing cells (*p* < 0.01) (Fig. 4*A*). Treatment also considerably enhanced IL-10 expression in different IL-10–expressing cell types, including CD4+ cells, CD8+ cells, B cells, dendritic cells, and macrophages, although the greatest contribution was derived from CD4+ T cells and macrophages (∼4.5-fold) (Figs. 4*B* and S4). Furthermore, compared with vehicle-treated EAE mice, MaR1-treated EAE mice had significantly greater plasma levels of IL-10 (*p* < 0.01) (Fig. 4*C*).

To test whether MaR1-induced IL-10 was responsible for the observed protective effects exerted by this proresolving mediator, we neutralized IL-10 with an anti-IL-10 neutralizing antibody (BioXCell) and found that, compared with IgG as a control, the anti-IL-10 antibody counteracted the protective effects of MaR1 in terms of the clinical score (Fig. 4*D*) and increased the infiltration of IL-17–producing CD4+ T cells into the CNS (Fig. 4*E* i-ii). Furthermore, to investigate whether IL-10 neutralization affects MaR1-induced loss of encephalitogenic characteristics in CD4+ cells, LN cells obtained at 21 dpi were grown under Th17 conditions as reported in Figure 4*B*, and on day 3, CD4+ T cells were adoptively transferred into naïve SJL mice, and the clinical score was assessed daily. As shown in Figure 4, *C* and *F*, the encephalitogenic properties of CD4+ T cells from MaR1+aIL-10-treated animals were restored to levels comparable to those of the EAE-Adt group. Taken together, these findings confirm that MaR1 mediates its protective effect *via* IL-10.

### MaR1 promotes metabolic reprogramming in effector CD4+ T cells and macrophages

**Figure 5:**
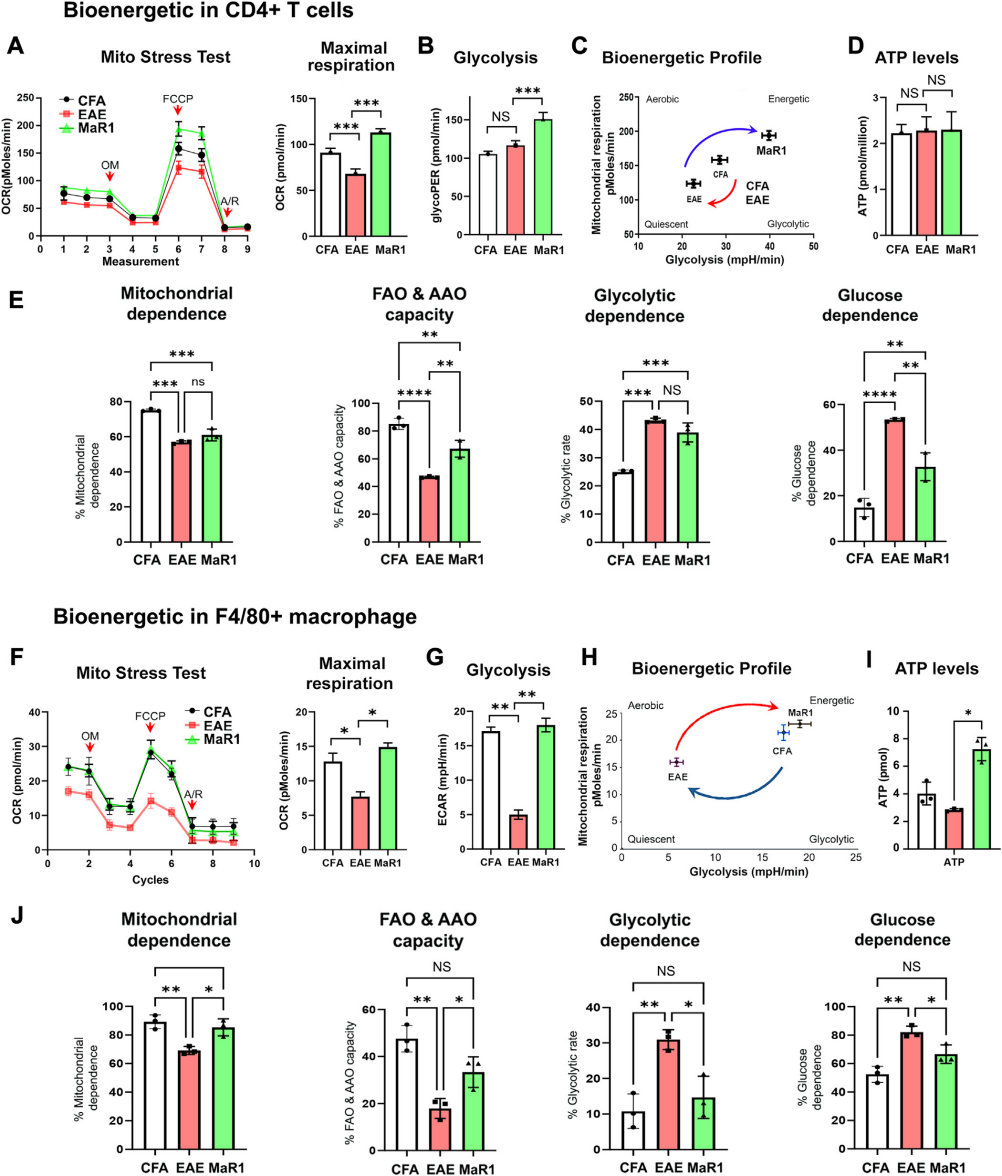
**MaR1 induces metabolic reprogramming in CD4+ T cells and macrophages of EAE mice.***A*, an XF mitochondrial stress test was performed on CD4+CD25 cells (purity ∼95%) isolated from CFA-, EAE-, and MaR1-treated EAE mice on day 18 postimmunization. The maximal respiration is presented as a bar graph (N = 6). *B*, compensatory glycolysis was examined using an XF Seahorse bioanalyzer and is presented as a bar graph (N = 6). *C*, the bioenergetic profile depicts the metabolic state of CD4+ cells isolated from the various groups described in (*A*). *D*, ATP levels detected in CD4+ cells from the various groups in (*A*) using an ATP assay kit (N = 4). NS, not significant compared with the CFA and EAE groups; Student’s *t* test was used. *E*, at the peak of the disease, brain infiltrating cells (BILs) were isolated from all groups using a Percoll gradient and processed for SCENITH. MFI of puromycin across samples treated with different inhibitors of CD4+ T cells (CD45^+^CD4^+^), including deoxyglucose (DG), oligomycin (OM), or deoxyglucose + oligomycin (DGO) (N = 3). Metabolic perturbations in infiltrating CD4+ cells from all mouse groups are shown as a bar graph. *F*–*I*, an XF mitochondrial stress test was performed on splenic F4/80+ macrophages (purity ∼95%) isolated from CFA-, EAE-, and MaR1-treated EAE mice. The maximal respiration, basal glycolysis, bioenergetic profile, and total ATP levels were detected in macrophages from all the mouse groups, as described above in detail. *J*, using SCENITH, metabolic perturbation of infiltrating macrophages (CD45^+^CD11b^+^F4/80^+^) from all the mouse groups was detected as described above. The data are shown as the mean ± SEM (N = 3). NS, not significant; ∗*p* < 0.05, ∗∗*p* < 0.01, ∗∗∗*p* < 0.001, ∗∗∗∗*p* < 0.0001 compared with CFA-treated or EAE mice, as determined *via* Student’s *t* test.

Since metabolic reprogramming is crucial for T-cell activation, differentiation, and effector function (33, 34), we next sought to investigate whether MaR1 has an impact on CD4 T-cell metabolism *in vivo*. CD4+ T cells (CD4+CD25-) isolated from MaR1-treated mice presented significantly greater mitochondrial respiratory capacity than did CD4+ T cells from vehicle-treated EAE mice (Fig. 5*A*). MaR1 improved the glycolytic rate in CD4+ T cells (Fig. 5*B*), resulting in a “higher metabolic state,” whereas vehicle-treated EAE CD4+ T cells presented a “lower metabolic state” (Fig. 5*C*); however, there was no change in total cellular ATP levels (Fig. 5*D*). These results suggest that MaR1 treatment affects the metabolic and bioenergetic states of CD4+ T cells *in vivo*.

To further investigate the effect of MaR1 on the metabolic fitness of infiltrated CD4+ T cells in the CNS, we used Single cell ENergetIc metabolism by profiling translation inHibition (SCENITH) to assess the metabolic state of numerous cell types *via* flow cytometry according to the gating strategy shown in Fig. S3. To do so, infiltrating mononuclear cells were isolated at 18 dpi, and single cells were treated with oligomycin, 2DG, or both (oligomycin+2DG), followed by a brief pulse of puromycin (PURO), allowing us to quantify metabolic fitness in terms of mitochondrial dependency, fatty acid and amino acid oxidation (FAO and AAO) capacity, and glycolytic and glucose dependence (35, 36, 37). We found that the infiltrated CD4 T cells from the MaR1-treated group had significantly greater mitochondrial dependency and FAO and AAO capability than did those from the vehicle-treated EAE group (Fig. 5*E*). Furthermore, MaR1 reduced glucose reliance in infiltrating CD4+ T cells without affecting glycolysis compared with that in infiltrating CD4+ T cells from vehicle-treated EAE mice (Fig. 5*E*).

Given that MaR1 treatment induced macrophage polarization toward an anti-inflammatory phenotype (Fig. 2, *F*–*H*) and that metabolic reprogramming plays a major role in macrophage phenotypic alterations (25, 38), we investigated the metabolic status of F4/80+ macrophages isolated from EAE mice treated with or without MaR1 at 18 dpi. Splenic macrophages isolated from the EAE group had significantly lower mitochondrial respiration and glycolysis than those of macrophages isolated from the CFA group, and these effects were reversed by MaR1 treatment (Fig. 5, *F* and *G*). Interestingly, macrophages from the CFA mice exhibited a higher metabolic state, whereas EAE macrophages exhibited a lower metabolic state, which was reversed by MaR1 treatment (Fig. 5*H*). Although there was no difference in total cellular ATP levels between the EAE and CFA groups, MaR1 significantly increased ATP levels in the treated EAE group compared to EAE and CFA groups (Fig. 5*I*), implying that an increased energetic state in macrophages in the MaR1 group may have contributed to energy production. Similar to CD4+ T cells, we investigated the metabolic fitness of F4/80 macrophages infiltrated by SCENITH according to the gating strategy shown in Fig. S1. Macrophages in the MaR1-treated mice had significantly greater mitochondrial dependency, FAO and AAO capability, and lower glucose and glycolytic dependence than those in the vehicle-treated EAE group (Fig. 5*J*). Overall, these results indicate that MaR1 treatment reshaped the metabolic and bioenergetic states of both peripheral and infiltrating CD4+ T lymphocytes and macrophages. However, the role of MaR1-induced metabolic reprogramming in altering the effector function of these immune cells has yet to be investigated.

### MaR1 enhances macrophage efferocytosis

**Figure 6:**
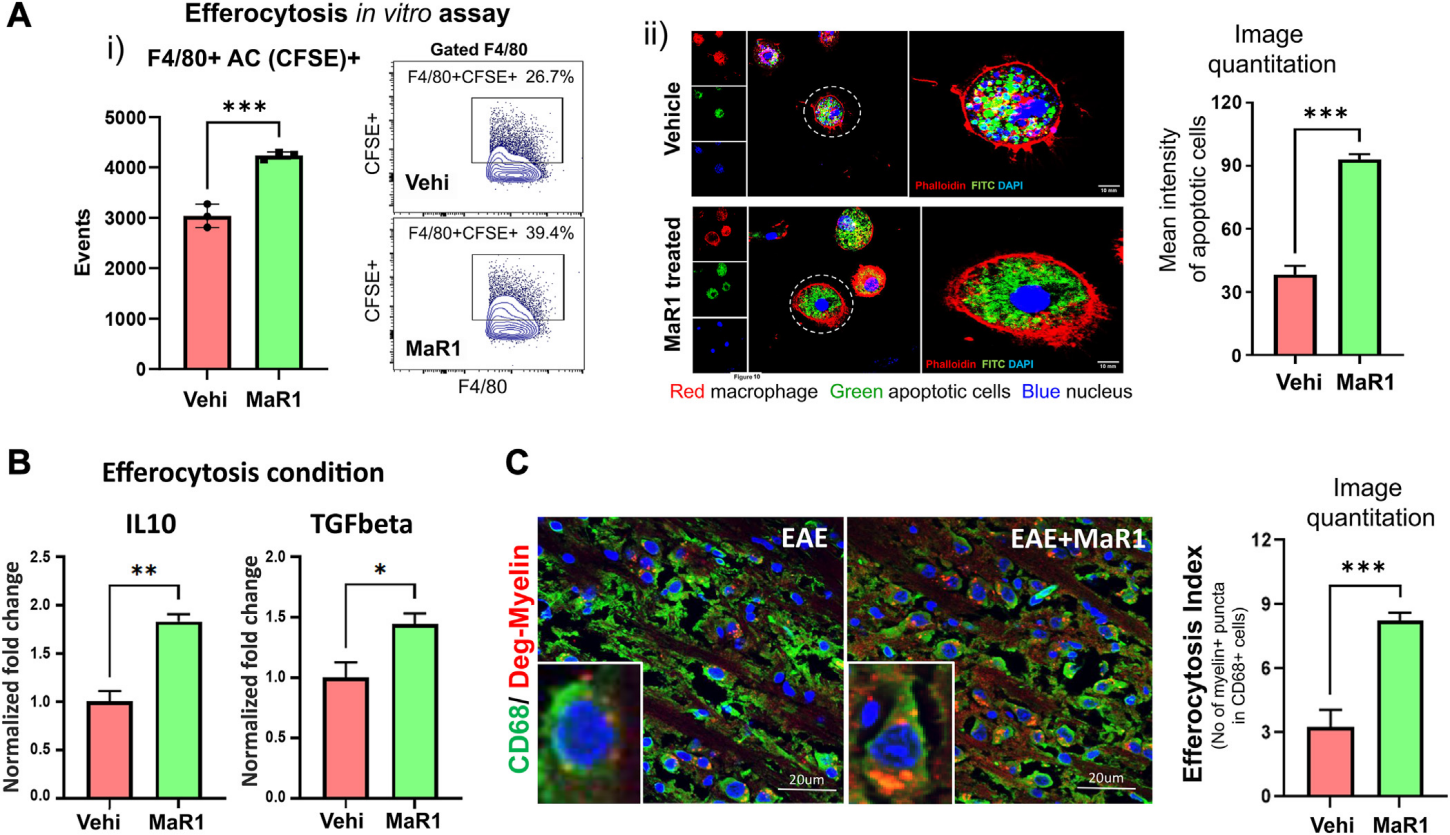
**MaR1 induces efferocytosis in macrophages.***A*i, bone marrow–derived macrophages were treated with MaR1 (100 nM) or vehicle (0.1% EtOH) for 1 h, after which CFSE-labeled 70 to 75% apoptotic splenic cells devoid of monocytes/macrophages were added at a ratio of 1:5. After 18 h, the cells were washed and stained for F4/80, after which the number of F4/80 cells engulfing CFSE-labeled cells was quantified (n = 3). The data are presented as a bar graph and a representative flow plot. *A*ii, another set of cells was seeded on coverslips, and an *in vitro* efferocytosis assay was performed as described above. After 18 h, the cells were washed and fixed, and Z-stack images were taken with an Olympus FV1000 confocal microscope with a 40 × objective. Four consecutive images with an interspace of 1 μm were captured. The mean intensity of apoptotic cells was quantified with ImageJ analysis software (version 1.49; NIH) (N = 6). *B*, under efferocytosis conditions, the expression of IL-10 and TGFβ was examined by qPCR, and the data were normalized to those of the housekeeping gene L27 (N = 3). *C*, for efferocytosis in the spinal cords of the EAE- and MaR1-treated groups, macrophages (CD68+) were stained with a mouse anti-CD68 antibody and polyclonal degraded myelin (Millipore). The images were captured by LSCM at a 1-ary unit aperture at 60x resolution (N = 5). The data are shown as the mean ± SEM. ∗*p* < 0.001, ∗∗*p* < 0.05, ∗∗∗*p* < 0.01.

The removal of dead cells by macrophages, termed "efferocytosis," is a key proresolving functional test (39, 40, 41), and many neurodegenerative disorders are caused by efferocytosis dysfunction (42). Using flow cytometry and confocal imaging, we found that MaR1-treated macrophages exhibited significantly higher efferocytosis than vehicle-treated macrophages did (Fig. 6*A*). Under efferocytosis conditions, MaR1 treatment increased the expression of the anti-inflammatory cytokines IL-10 and TGFβ (Fig. 6*B*). Furthermore, we observed a similar impact of MaR1 *in vivo* by analyzing brain sections and observing that spinal cord CD68+ macrophages from MaR1-treated EAE mice presented a greater efferocytosis index for myelin debris than did those from vehicle-treated EAE mice (Fig. 6*C*). These findings show that MaR1 promotes macrophage efferocytosis both *in vitro* and *in vivo*.

### MaR1 induces metabolic reprogramming in microglia by switching them to an anti-inflammatory state

**Figure 7:**
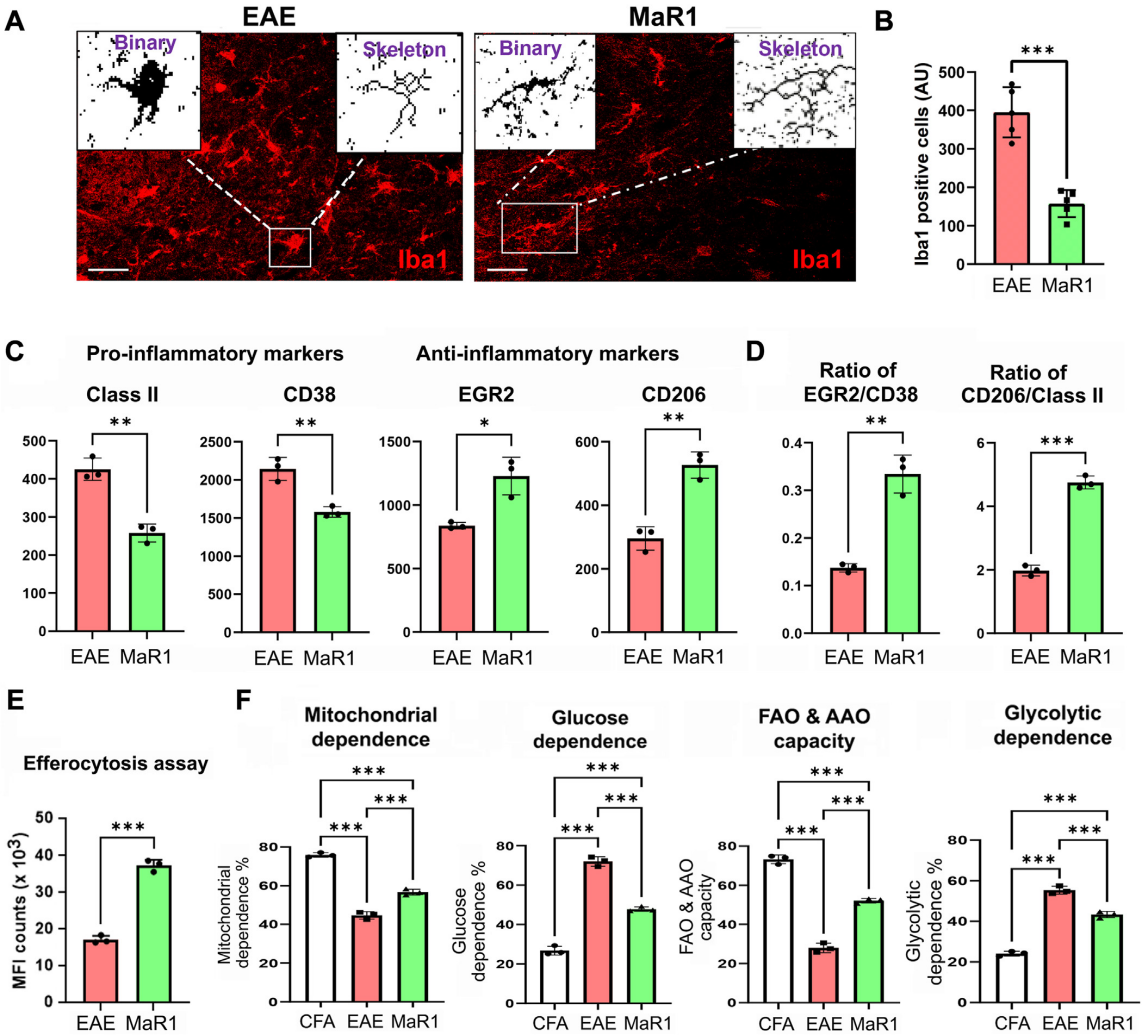
**MaR1 promotes an anti-inflammatory phenotype in microglia.***A*, to examine microglial morphology in response to MaR1 treatment, lumbar spinal cord sections from the EAE- and MaR1-treated groups were stained with Iba1, and immunofluorescence images were threshold filtered, binarized, and analyzed *via* ImageJ/FIJI software for skeletal analysis (n = 5). Scale bar represents 100 μm. *B*, quantification of Iba1-immunoreactivity–positive cells is presented in a bar graph (n = 5). *C*, at the peak of the disease, after ∼18 days, brain infiltrating leukocytes (BILs) from all groups (EAE- and MaR1-treated) were processed for pro- and anti-inflammatory marker detection *via* flow cytometry (n = 3). *D*, bar graph of the EGR2/CD38 and CD206/class II ratios. *E*, primary microglia were treated with MaR1 (100 nM) or vehicle (0.1% EtOH) for 1 h, after which CFSE-labeled 70 to 75% apoptotic splenic cells devoid of monocytes/macrophages were added at a ratio of 1:5. After 18 h, the cells were washed and stained for CD11b, after which the number of CD11b + microglia engulfing CFSE-labeled cells was quantified (n = 3). The data are presented as a bar graph. *F*, changes in microglial metabolism in all groups were evaluated *via* SCENITH, and the data are presented in a bar graph as the mean ± SEM (n = 3). NS, not significant; ∗*p* < 0.05, ∗∗*p* < 0.01, ∗∗∗*p* < 0.001 compared with EAE- versus MaR1-treated EAE.

Microglia, or brain-resident macrophages, can play two different roles in neuroinflammation (43). Depending on the environment, microglia can be either protective or detrimental to neurodegeneration, polarizing them into pro- or anti-inflammatory states. At the same time, they undergo distinct morphological changes during activation (44). In the lumbar area of the spinal cord in EAE mice, activated microglia exhibited shorter branches, an enlarged soma, and a less ramified structure; however, in the MaR1-treated group, microglia appeared less activated and displayed fine, elongated branches, a normal soma, and a more ramified morphology (Fig. 7*A*). Moreover, MaR1 treatment significantly reduced the number of Iba1-immunoreactive cells compared with that in the EAE group (Fig. 7*B*), indicating that MaR1 treatment reduced the activation of microglia and maintained them in a ramified form in the treated group. To further strengthen our observation, we examined the phenotype of microglia using flow cytometry. We observed that MaR1 treatment decreased the expression of the pro-inflammatory markers MHC-II and CD38 while increasing the expression of the anti-inflammatory markers EGR2 and CD206 (Fig. 7*C*). The ratios of pro- versus anti-inflammatory markers (EGR2/CD38 and CD206/class II) (Fig. 7*D*) demonstrated that MaR1 promotes the anti-inflammatory phenotype. Like other macrophages, MaR1 also significantly induced efferocytosis in primary microglia (Fig. 7*E*), as microglia are professional CNS phagocytes that engulf myelin debris and initiate synaptogenesis and neurogenesis (45). We recently used an EAE model to study the metabolic control of microglia during neuroinflammation *in vitro* and *in vivo* (37). Thus, we investigated whether MaR1 treatment reversed metabolic perturbations in microglia in the EAE group. Similar to our previous work (37) utilizing SCENITH and flow cytometry (Fig. S3), we observed that microglia in the EAE group had lower mitochondrial function and greater glycolysis than those in the CFA control group did, which was reversed by MaR1 treatment (Fig. 7*F*). These findings demonstrate that MaR1 induces an anti-inflammatory phenotype in microglia, which may contribute to the proresolving effects of MaR1 on EAE possibly *via* metabolic reprogramming.

### MaR1 promotes oligodendrocyte metabolic fitness during EAE

**Figure 8:**
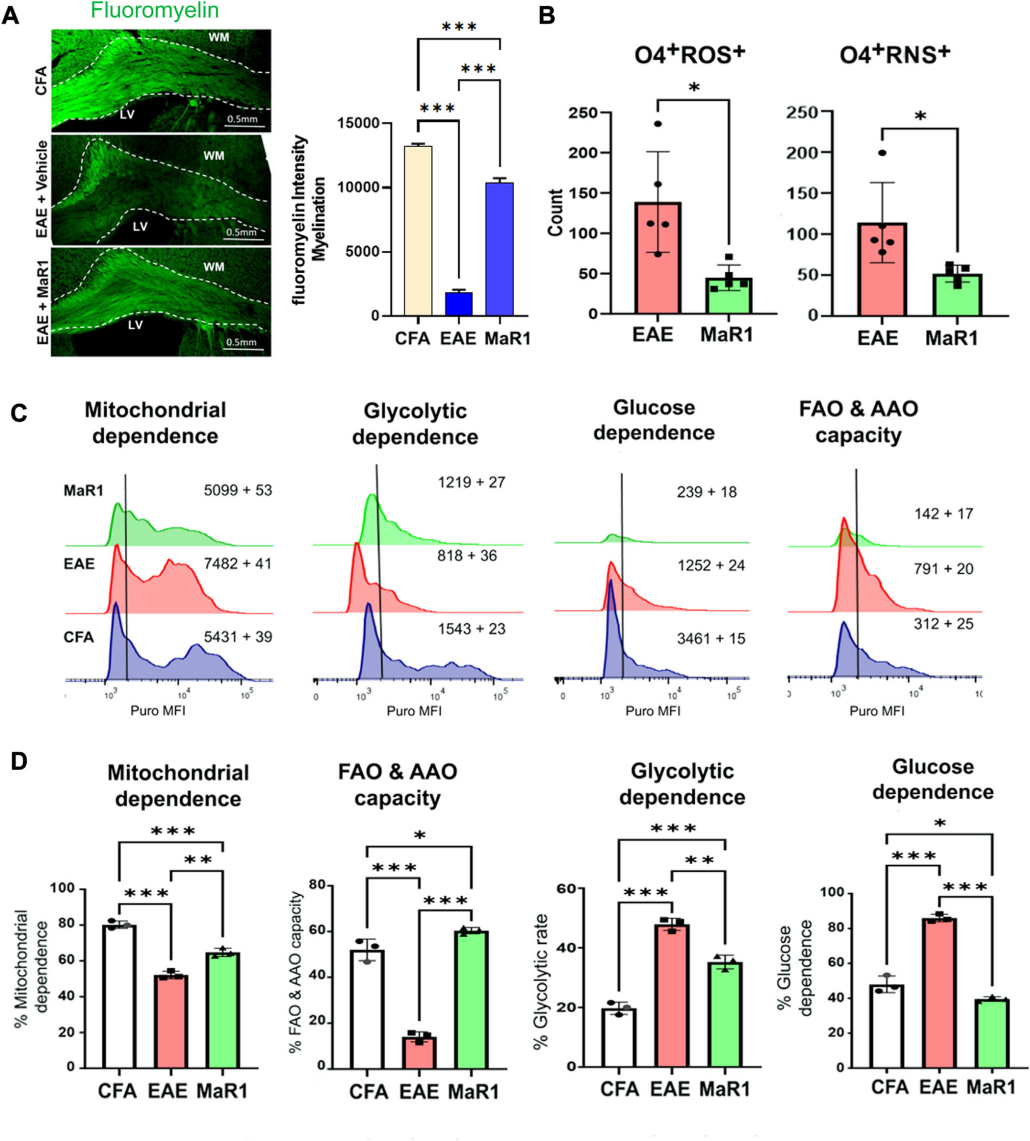
**MaR1 protects myelin possibly by regulating the metabolic fitness of oligodendrocytes during EAE.***A*, serial coronal brain sections from CFA-, EAE-, and MaR1-treated EAE mice on day 18 postimmunization were stained with fluoromyelin, and imaging was performed *via* laser scanning confocal microscopy. The intensity of myelin in the corpus callosum (CC) above the lateral ventricle was measured with ImageJ (labeled LV) (N = 3). *B*, at the peak of the disease, single suspensions of CNS tissues were processed *via* a Percoll gradient from all groups (CFA, EAE, and MaR1-treated), and the isolated cells were processed for the detection of intracellular reactive oxygen and nitrogen species (ROS and RNS) levels in O4+ oligodendrocytes (CD45^-^O4^+^), which were evaluated *via* the Cellular ROS/RNS Detection Assay Kit *via* flow cytometry (n = 5). *C* and *D*, puromycin incorporation (MFI values indicated) in O4+ oligodendrocytes (CD45^-^O4^+^) in EAE- and MaR1-treated EAE mice. Metabolic changes, including changes in mitochondria, glycolysis, and fatty acid oxidation (FAO), in O4^+^ oligodendrocytes in all groups were calculated *via* SCENITH (N = 3) and are shown as a bar graph. ∗*p* < 0.05, ∗∗*p* < 0.01, ∗∗∗*p* < 0.001 compared with CFA or EAE.

Since MaR1 decreased myelin loss, as shown by Luxol fast blue staining (Fig. 4*C*), we investigated whether this effect was made possible by healthy, metabolically fit oligodendrocytes. Representative immunofluorescence images confirmed greater myelin basic protein expression in the white matter (corpus callosum) of the brains of MaR1-treated EAE mice than in those of the vehicle-treated EAE mice (Fig. 8*A*). Furthermore, MaR1 treatment reduced the levels of ROS and RNS in O4+ oligodendrocyte populations (Fig. 8*B*). The metabolic parameters of these cells were investigated using SCENITH, flow cytometry, and PURO MFI (Fig. 8*C*). This analysis revealed that O4+ oligodendrocytes from EAE mice were glycolysis-dependent and exhibited a significant reduction in mitochondrial respiration and FAO, which were restored by MaR1 treatment (Fig. 8*D*). We also observed a significantly higher number of oligodendrocyte progenitor cells (OPCs [Pdgfra + cells]) and premyelinating-oligodendrocytes (O4+ cells) in the MaR1-treated spinal cord than in the vehicle-treated EAE spinal cord (Fig. S5), indicating that MaR1-mediated protection against demyelination could culminate in neuroprotection and/or remyelination, which needs to be investigated in detail. Overall, these findings suggest that MaR1 may protect against demyelination and restore dysfunctional oligodendrocyte metabolism.

### MaR1 reverses EAE-induced alterations in the spinal cord transcriptome

**Figure 9:**
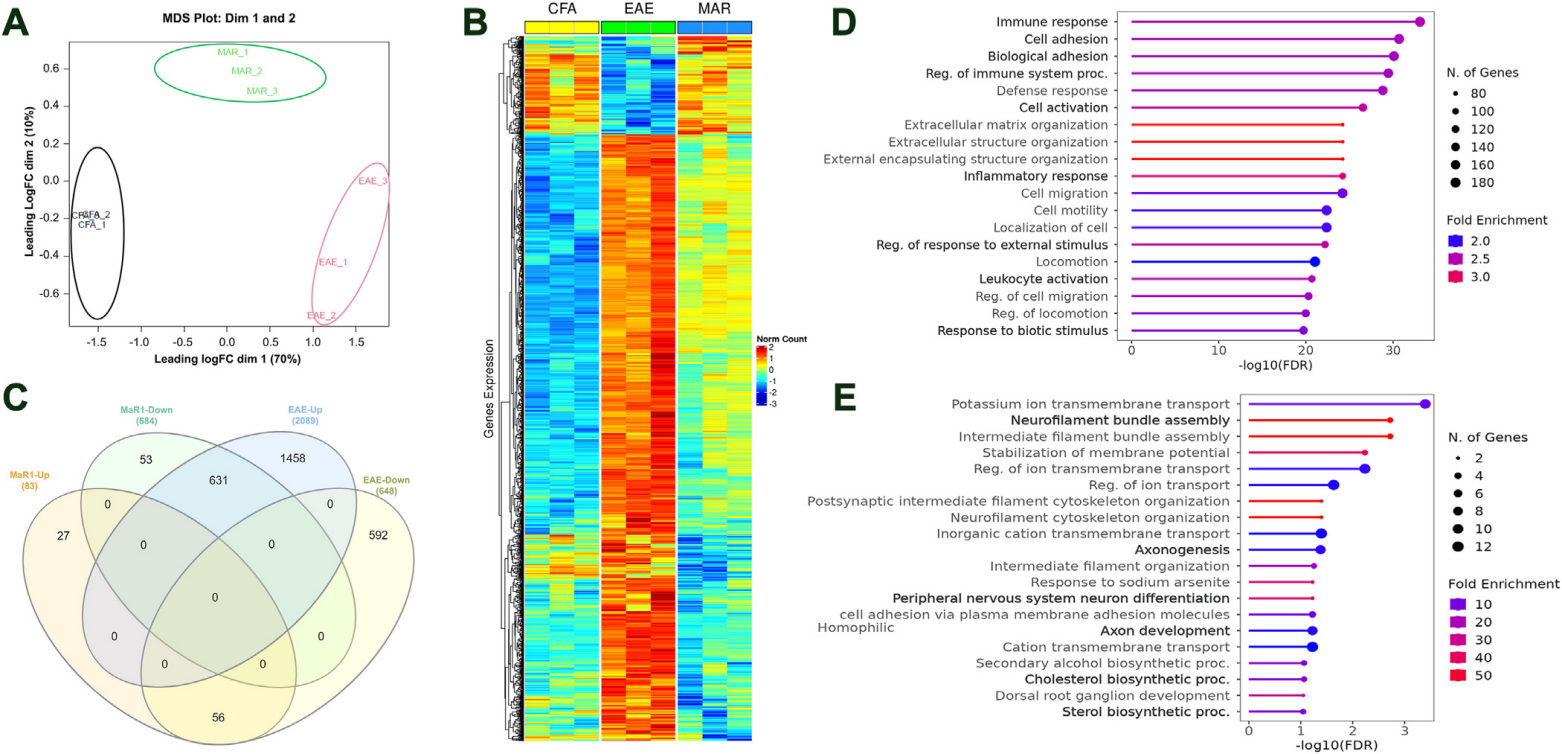
**MaR1 rescues EAE-induced spinal cord transcriptomic alterations.***A*, Multidimensional scaling (MDS) plot showing the segregation of each group: CFA, EAE, and MaR1. *B*, heatmap representing significant gene expression changes across the groups. Padj>0.05. *C*, Venn diagram representing the shared number of upregulated and downregulated genes in MaR1-treated EAE mice. *D*, gene ontology (GO) enrichment of common genes upregulated by EAE and downregulated by MaR1 treatment. FDR<0.05. *E*, gene ontology (GO) enrichment of common genes downregulated by EAE and upregulated by MaR1 treatment. FDR<0.05.

Since MaR1 exerts several protective effects at different levels, involving different cell types, we investigated whether transcriptome changes were associated with MaR1 treatment by performing RNA-seq on lumbar spinal cord tissue from CFA-, EAE-, and MaR1-treated mice 70 dpi, namely, after the RR course of the disease. Interestingly, we found a clear distinction between these groups (Fig. 9*A*), with numerous differentially expressed genes. Overall, 631 genes were significantly upregulated in the untreated EAE mice but downregulated in the MaR1-treated EAE mice (Fig. 9, *B* and *C*). With respect to the functional relationships of the dysregulated genes, according to Gene Ontology (GO) analysis, the downregulated genes were associated with the immune response, cell and biological adhesion, cell activation, leukocyte activation, and other immune/defense-related pathways (Fig. 9*D*). Furthermore, MaR1 dramatically increased the expression of a limited subset of genes involved in axonogenesis and related functions and cholesterol biosynthetic pathways, the majority of which were downregulated in EAE (Fig. 9*E*). Notably, MaR1 treatment did not exacerbate any gene dysregulation caused by EAE, suggesting that MaR1 helps prevent EAE-induced inflammation and may even concomitantly drive myelination and axonal regeneration.

### MaR1 reduces T-cell autoreactive responses and promotes IL-10–producing Tregs in RR-MS patients

**Figure 10:**
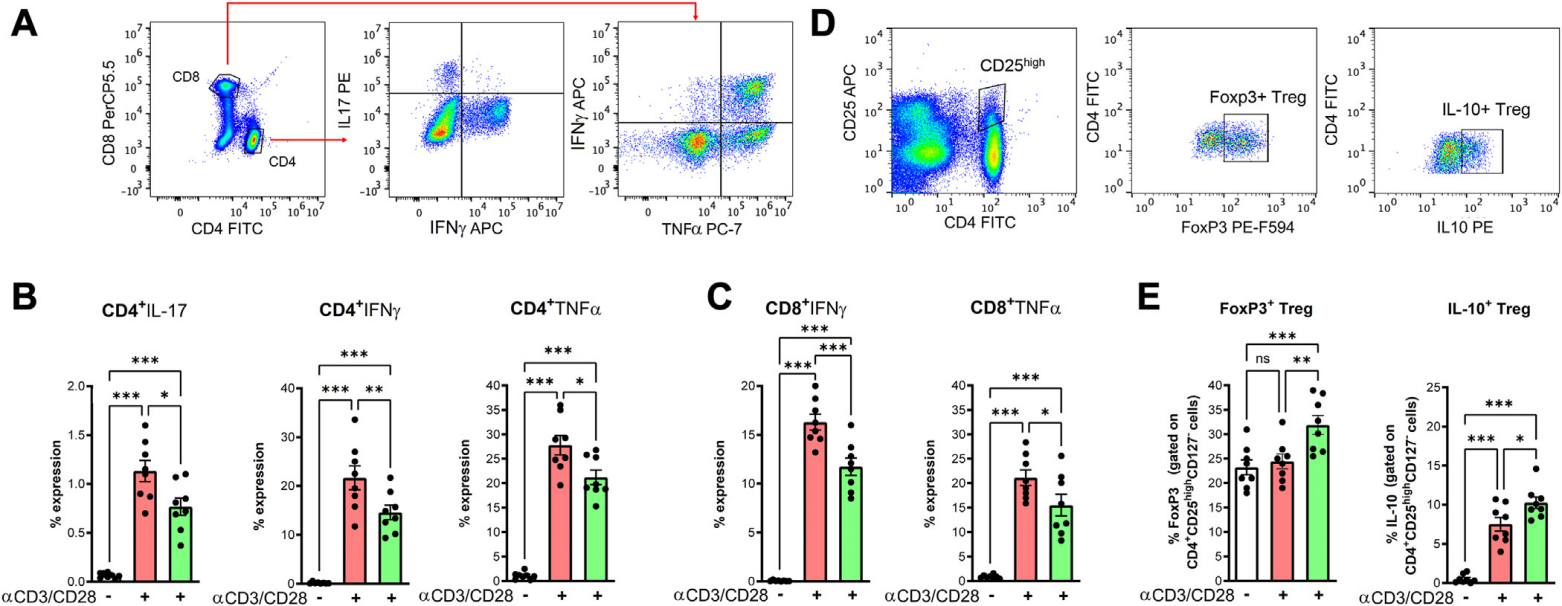
**MaR1 reduces cytokine responses in activated human T-cell subsets in patients with relapsing-remitting (RR)-MS.***A* and *D*, peripheral blood mononuclear cells (1 × 10^6^ cells per well) were *left* untreated or treated with vehicle or MaR1 (10 nM) for 30 min (N = 8). The cells were then stimulated with anti-CD3/CD28 for 8 h, stained both at the cell surface and intracellularly, and analyzed by flow cytometry. *B* and *C*, cytofluorimetric plots and percentages of intracellular IFN-γ and IL-17 produced by CD4^+^ T cells and of TNF-α and IFN-γ produced by CD8^+^ T cells. *E*, cytofluorometric plots and percentages of intracellular FoxP3 and IL-10 in Tregs. NS not significant, ∗*p* < 0.05, ∗∗*p* < 0.01, ∗∗∗*p* < 0.001.

To translate these findings to the human setting, we investigated whether MaR1 could also impact human T-cell activity. For this purpose, we treated human T cells from patients with RR MS (Table 1). In particular, the immunomodulatory role of MaR1 was investigated by inducing polyclonal activation of the T-cell receptor with anti-CD3 and anti-CD28 antibodies. The effect of MaR1 on specific T-cell subsets was observed in CD4+ Th1 and Th17 cells, as well as in cytotoxic CD8+ T cells. As expected, and as previously shown (46), anti-CD3 and anti-CD28 stimulation resulted in high percentages of TNF-α–, IFN-γ–, and IL-17–producing T cells in all T-cell subsets (Fig. 10, *A*–*C*). MaR1 treatment significantly impacted all T-cell subsets by significantly halting IFN-γ and IL-17 production (CD4+ T cells) (Fig. 10*B*) and by reducing TNF-α and IFN-γ production (CD8+ T cells) (Fig. 10*C*). Moreover, MaR1 potentiated Treg responses by significantly increasing the expression of FoxP3, a signature transcription factor, and the production of IL-10 (Fig. 10, *D* and *E*). Our data on patient-derived PBMCs substantiate the protective role of MaR1, expanding the therapeutic potential of SPMs.

**Table 1:** Demographic data of RR-MS patients

Characteristic	RR-MS
No of subjects	N = 8
Mean age (years)	34.10 ± 8.72
Female/male	5/3
Disease duration (years)	6.4
Relapse/Remission	4/4
Mean EDSS	1.8
DMT when sampling (yes/no)	0/0
Corticosteroids (yes/no) (>1 month before sampling)	0/0

## Discussion

Despite the availability of numerous immunomodulatory drugs, there are no MS treatments that are entirely satisfactory, as most drugs have substantial side effects, with less-than-optimal tolerance, and do not provide complete remission or disease stabilization. Given that unresolved inflammation is a hallmark of MS pathogenesis, it is imperative to study the endogenous mechanisms that curtail excessive inflammation and encourage timely resolution. In MS, disease progression may be partly consequential for the failure of inflammation resolution, which is associated with metabolic dysfunction and mediated by resolution mediators (12). However, there is a gap in knowledge regarding the role of endogenous resolution mediators in MS. Identifying a therapeutic option that can modulate adaptive and innate immune responses without generally suppressing the immune system has been a key barrier to improving treatment for patients with MS. With metabolic profiling, we have demonstrated in our previous work that the precursor of MaR1 (14-HDHA), a proresolving lipid metabolite of omega-3 PUFAs, is significantly lower in the plasma of patients with MS (13).

A recent study showed that MaR1 has a protective effect in a chronic model of EAE (47). However, its impact on RR-EAE and the underlying in-depth mechanisms have not been reported. In this study, we investigated the impact of MaR1 on inflammation and disease severity in an RR-EAE model and investigated how MaR1 treatment polarized adaptive and innate immune cells to dampen the antigen-specific immune response in this RR-EAE model. Our findings indicate that administering MaR1 at a dosage of 300 ng/mouse on day 6 after immunization or during the remission of clinical symptoms significantly reduces symptom severity in EAE models. The results presented here provide evidence for the potential use of maresins in clinical settings. This finding is particularly significant because maresins do not suppress the immune system (48), unlike steroids and many other treatments for MS. These findings suggest that MaR1 could be a new and powerful therapeutic alternative for MS that does not have immunosuppressive effects. Nevertheless, the administration of MaR1 reduced the production of IL-17 and stimulated the production of IL-4 and IL-10. These results emphasize the immunomodulatory function of MaR1 in EAE by reducing pro-inflammatory conditions in the CNS and adjusting T-cell responses to promote an anti-inflammatory state. However, the beneficial effects of MaR1 on reducing neuroinflammation in EAE are substantial, as MaR1 significantly protects against neurological impairment and myelin loss, even when treatment is initiated on day 6 after immunization. Overall, our findings indicate that MaR1 expedited the resolution of inflammation in EAE and effectively halted the course of the disease. Additionally, MaR1 has been implicated in various disease models, such as spinal cord injury (15), spinal muscular atrophy (49), and cerebral ischemia (17). The proresolving role of MaR1 in macrophages is facilitated by its interaction with its receptor, G-protein coupled receptor 6 (*Lgr*6) (50). However, its impact on CD4+ cells has not been studied. *Lgr*6 expression has been observed in regulatory T cells and has been found to facilitate the protective impact of MaR1 in a model of respiratory viral infection (51). Nevertheless, the process by which the MaR1-*Lgr*6 cascade influences the progression of EAE disease has not yet been examined.

Several studies have demonstrated the role of DHA in protecting the brain, but the specific processes involved are not completely understood. These mechanisms may include maintenance of the myelin sheath, which helps preserve the integrity of axons and hence facilitates the process of remyelination in brain diseases (52, 53). DHA-derived metabolites possess neuritogenic, synaptogenic, and neurogenic characteristics. Studies have demonstrated that treatment with DHA, as well as certain metabolites produced from it, can improve or resolve inflammation, thereby serving as a protective nutritional shield for the brain (52, 54, 55, 56, 57, 58, 59). In the past, our group reported the very first study indicating the therapeutic potential of a specific SPM, resolvin D1 (RvD1). The effects of this SPM on disease progression were demonstrated in chronic and RR-EAE models (60). We reported that RvD1 suppressed EAE disease progression by suppressing autoreactive T cells and inducing an anti-inflammatory phenotype in macrophages (60). Additionally, a more recent publication provided further evidence of the beneficial effects of DHA metabolites, including RvD1 and protectin D1. In this study, these two metabolites prevented inflammation-induced dysfunction of the blood‒brain barrier (14). Taken together, these studies offer vital information regarding the therapeutic capacity of metabolites produced from DHA for the treatment of MS.

Our research revealed that MaR1 functions as an immunomodulator, as indicated by changes in the immune system of EAE mice within the central nervous system. Unlike the results reported by Sánchez Fernández *et al.* (47), MaR1 significantly increased the RNA and protein levels of anti-inflammatory cytokines, specifically IL-4 and IL-10, which could have mouse strain–specific effects. This led to the promotion of an anti-inflammatory state in CD4 T cells and a decrease in the number of pathogenic CD4+ cells that produce IL-17. Since IL-10 plays a crucial role in the pathogenesis of MS and other neurological illnesses (61), any molecule, such as MaR1, that increases the production of IL-10 has significant potential for therapeutic improvement. Furthermore, MaR1 reduced the pathogenic response of T cells in MS patient-derived PBMCs, indicating its protective and translational role in MS. Tregs expressing IL10 post-MaR1 treatment play a role in modulating the T-cell response, indicating its anti-inflammatory role in MS. However, mechanistic studies are needed to decipher the signaling pathway through which MaR1 plays a pleotropic role in mitigating inflammation in chronic inflammatory diseases such as MS.

Efferocytosis is crucial for the recovery of homeostasis of the brain parenchyma not only because it removes apoptotic cells before they progress to secondary necrosis, which releases toxic intracellular compounds and autoantigens but also because it modulates the phagocyte inflammatory response (62, 63). Efferocytes are primarily monocytes/macrophages and brain-resident macrophages (microglia) (64). Failed efferocytosis is emerging as a key mechanism driving the progression of chronic inflammatory diseases, including metabolic diseases and neurodegenerative diseases (42, 65). It is impaired in several pathophysiological processes, giving rise to chronic inflammatory and neurodegenerative diseases such as MS (66, 67). CNS diseases share some cardinal neuropathological events, such as inflammation and excitotoxicity, thus raising the possibility of phagocytosis impairment during the disease course (68, 69). There is a lack of understanding of how efferocytosis plays a protective role during MS. Efferocytosis relies on metabolic signaling to stimulate anti-inflammatory responses (70, 71), and pharmacological manipulation of these metabolic pathways can be critical for promoting repair and healing in the body (72). We observed that MaR1 restored metabolic reprogramming in macrophages/microglia, which could be one of the potential mechanisms of MaR1-mediated reversal of impaired efferocytosis in EAE. Moreover, its treatment induced the production of IL-10 and TGFbeta, which are anti-inflammatory cytokines that protect against EAE under efferocytosis conditions. Additional in-depth studies are needed to elucidate the mechanism by which MaR1 controls efferocytosis in EAE models. This could reveal new concepts and unexplored possibilities for the development of innovative treatments that enhance the healing and rejuvenation of the CNS, not only for MS but also for other neurological disorders.

MaR1 can also protect microglia by supporting an anti-inflammatory state and initiating metabolic reprogramming. These findings indicate that microglia play a role in restoring the CNS microenvironment toward the resolution phase in EAE. Several studies have reported the protective role of microglia against EAE pathology (73, 74, 75). Microglia also play a critical role in remyelination processes (76). The increased anti-inflammatory phenotype of microglia and myelin protection in MaR1-treated EAE mice suggests the possibility of interplay between microglia and oligodendrocytes. Importantly, MaR1 maintains myelin status by inducing metabolic changes and reducing oxidative stress in oligodendrocytes, the myelin-producing cells of the CNS, suggesting a possible link between its neuroprotective effects and metabolic reprogramming of disease-associated cell types. Abnormal metabolic pathways in oligodendrocytes have been linked to a variety of neurodegenerative disorders (77, 78). We, and others, have demonstrated that metabolism is critical for the maturation of oligodendrocyte precursor cells into preoligodendrocytes (79) and for the shift from preoligodendrocytes to mature oligodendrocytes (80). Given the critical role of oligodendrocytes in remyelination, limited information is available concerning MaR1-induced metabolic changes during oligodendrocyte development and how these changes contribute to the maintenance of myelination status. However, further studies are needed to establish whether the effects of MaR1 on neuroprotection and metabolism could be secondary to its anti-inflammatory effect or whether it could exert direct effects on OPCs and on premyelinating-oligodendrocytes to induce myelination.

## Conclusion

Our results demonstrate that MaR1 has a broad effect on the functional capabilities of immune cells associated with EAE and MS, resulting in decreased severity and progression, as well as improved neurological outcomes in RR-EAE model of MS. However, further studies are necessary to determine the role of MaR1 in regulating immune cell metabolism and how it impacts effector functions and disease outcomes.

## Experimental procedures

### Ethical approval

The animal studies performed in this manuscript were approved by the IACUC committee of Henry Ford Health (IACUC protocol # 1419). Human studies reported in our manuscript abide by the Declaration of Helsinki principles. All human subjects provided informed written consent for the study, which was approved by the ethics committee of IRCCS Neuromed (protocol 26.03.2018).

### EAE induction and functional evaluation

Female 10- to 12-week-old SJL mice were purchased from the Jackson Laboratory. The animals were housed in a pathogen-free animal facility of Henry Ford Health, Detroit, MI, in accordance with the Animal Care and Use Committee of Henry Ford Hospital. On day 0, each mouse was immunized by subcutaneous flank region injection with 200 μg of the antigen, proteolipid protein_139-151_ (PLP_139-151_) peptide, emulsified in 200 μl of Complete Freund’s Adjuvant (CFA, Sigma Chemicals) supplemented with 4 mg/ml heat-killed *Mycobacterium tuberculosis* H37Ra (400 μg; Becton, Dickinson and Company) as described previously (25, 60, 81). One set of mice was injected with CFA without antigen to serve as a control. All the mice were housed with standard food and water ad libitum at room temperature (22 ± 2 °C) under a 12:12 h light:dark cycle. Clinical scores were monitored daily until the duration of the study in a blinded fashion by measuring paralysis according to the conventional grading system as described previously (25, 60, 81). EAE mice treated with PBS or MaR1 were euthanized as indicated in the figures. SJL mice were anesthetized with CO_2_ and transcardially perfused with 1 × chilled PBS, followed by perfusion with 4% paraformaldehyde for histopathological analysis and confocal imaging.

All animal experiments in this study were performed according to the policies and guidelines of the IACUC committee at Henry Ford Health under the animal welfare assurance number D16-00090.

### MaR1 administration

The mice were randomized into treatment and control experimental groups (CFA, EAE, and EAE treated with MaR1; hereafter referred to as MaR1). The MaR1 solution was freshly prepared from a stock solution stored in a deep freezer and administered immediately to prevent degradation. After disease induction, daily intraperitoneal injections (i.p.) of 200 μl of PBS, 300 ng of MaR1 (7R,14S-dihydroxy-4Z, 8E, 10E, 12Z, 16Z, or 19Z-DHA; Cayman Chemicals) per mouse, or vehicle (0.1% ethanol), were administered starting from day 6 until the end of the study.

For the IL-10–neutralizing experiments, SJL mice were immunized as described above (for EAE induction and functional evaluation), followed by i.p. injection of 200 μg of anti-IL-10 (BioXCell # BE0049) twice a week concurrently with daily MaR1 i.p. treatment (300 ng/mouse). The control mice received isotype-matched rat IgG1 (BioXCell # BE0088) following the same protocol. Clinical symptoms were recorded as the clinical score, and the mice were subsequently euthanized at 21 days postimmunization (dpi).

### Behavioral test

Locomotor activity was measured to assess the effect of MaR1 treatment on EAE. We employed an infrared-based technology called the novel infrared-based automated activity monitoring system (21), developed by Columbus Instruments International. This instrument is supported by several electrodes embedded in an electric sensing board spaced 4 inches apart, with a beam diameter of 0.125 inches and a beam scan rate of 160 Hz. The sensors were properly screwed on a bracket to increase the flexibility and adjustability of the device. Acrylic cages were placed between the brackets. Locomotor activity was measured in the form of diurnal horizontal (X-axis) and nocturnal vertical (Z-axis) movements, and quantitative analysis was performed with MDI software (Columbus Instruments International). This quantitative analysis was performed by calculating the number of infrared interruptions emitted by the mice at specific time intervals in the system.

### Antigen recall response

For the antigen recall response, spleen and LN cells (4 × 10^6^/ml) were cultured in the presence or absence of the PLP_139-151_ antigen (20 μg/ml). The production of pro- and anti-inflammatory cytokines (IFNγ, GM-CSF, IL-17a, IL-10, and IL-4) was observed as described previously (25, 60, 81, 82).

### Macrophage and CD4+ cell isolation

On day 18 postimmunization, F4/80 macrophages were isolated from the spleens of EAE mice, treated with or without MaR1, using a Miltenyi Biotec Kit (#130-110-443; ∼92% purity). CD4+ cells were also isolated from spleens using a CD4 isolation kit (BioLegend # 480033; ∼94% purity).

### Adoptive transfer

For adoptive transfer experiments, EAE was induced in SJL mice, and MaR1 treatment began when the randomized mice had recovered from the disease. At the end of the study, LNs were isolated from both MaR1-treated and untreated EAE mice and cultured with PLP_139-151_ (20 μg/ml), anti-IFNy (10 μg/ml), IL-12p70, and IL-23 (10 ng/ml). After 3 days of incubation, 5 million CD4+ cells were injected intraperitoneally into SJL mice (n = 5), followed by daily evaluation of the clinical score.

### SiMoA assay

To assess the effect of MaR1 on the CNS in EAE mice, we measured plasma neural marker levels (NFL) in MaR1-treated and untreated EAE mice *via* the commercially available SiMoA Neuro 2-Plex B Advantage Kit (product number: 103,520) (Quanterix) on an SR-X analyzer according to the manufacturer's instructions. Additionally, the effect on inflammation was examined *via* cytokine profiling. We used a Mouse Cytokine 5-Plex Kit for IL-6, TNFα, IL-17, IL-12, and IL-10 (Cat # 107-178-1-AB; Product Number: 85-0441) (Quanterix) on an SP-X analyzer according to the manufacturer's instructions. SiMoA was chosen for this experiment since it is an emerging ultrasensitive technology with the potential to detect analytes at levels below the detection limits for other conventional assays.

### RNA isolation and qPCR

Total RNA was isolated from cells with the QIAzol reagent in the RNeasy kit (Qiagen). For cDNA synthesis, 1 μg of RNA was used *via* an iScript cDNA synthesis kit (Bio-Rad) according to the manufacturer’s guidelines. qPCR analysis was performed using SYBR Green (Bio-Rad) on a Bio-Rad CFX96 real-time PCR detection system with accompanying software (CFX Maestro, Bio-Rad). Gene expression was normalized to that of the control housekeeping gene, ribosomal L27, using CFX Maestro software (Bio-Rad).

### Enzyme-linked immunosorbent assay

The protein levels of the cytokines IFN-γ, IL-17A, GMCSF, IL-4, and IL-10 were quantified *via* standard ELISA kits provided by Biolegend and BD Biosciences, according to the manufacturer’s instructions.

### Immunohistology

Briefly, brain and spinal cord tissues were harvested from euthanized animals with CO2 and postfixed in 4% paraformaldehyde at 4 °C for 48 h, followed by the addition of a cryoprotectant with 30% sucrose until the tissue sank. The tissues were subsequently embedded in optimal cutting temperature compound, and the frozen blocks were sliced into sections (5–10 μm) to assess the infiltration of immune cells and demyelination in the CNS. Histopathological evaluations *via* H&E and Luxol fast blue staining methods were performed as described previously (25, 82). For microglia, OPC, and premyelinating oligodendrocytes staining, lumbar sections of the spinal cord were deparaffinized and rehydrated through a graded alcohol series, followed by heat-mediated antigen retrieval. The sections were subsequently blocked with 1% bovine serum albumin at room temperature followed by overnight incubation at 4 °C with the following primary antibodies: anti-rabbit Iba1 (1:200, ab178846), anti-rat PDGFRα (1:250, Biolegend, APA5), and anti-mouse O4 (1:250, MAB345). For myelin staining, frozen sections were washed for 20 min with 0.1% PBS-Triton X-100, incubated with a blocking solution containing 1% bovine serum albumin for 1 h at room temperature, and then incubated with a mouse anti-myelin basic protein monoclonal antibody (1:500; Cell Signaling Technology) overnight at 4 °C. The sections were subsequently washed and incubated with Alexa Fluor 568- or Alexa Fluor 488-coupled secondary antibodies (Life Technologies). Nuclei were counterstained with 4′,6-diamidino-2-phenylindole (H-1200; Vector Laboratories, Inc.) and cover slipped. Images were captured with a laser scanning confocal microscope (Olympus) and then quantified *via* ImageJ (National Institutes of Health).

### Microglial morphology analysis

Microglial morphology was analyzed *via* skeleton analysis. Iba1-stained images were scanned and converted into binary images to evaluate the cytoskeletal architecture of microglia. The ImageJ/FIJI software, which offers the skeleton analysis (2D/3D) plugin, was employed to provide detailed information on the microglial population.

### Flow cytometry

Immune cell profiles in the spleen/LNs and CNS (brain and spinal cord) of EAE mice were assessed at different time points for the corresponding experiments *via* standard staining methods and analysis protocols as previously described (25, 60, 82). All the data were processed *via* FlowJo software and an analysis program (Treestar).

### Detection of ROS/RNS

The intracellular levels of reactive oxygen and nitrogen species (ROS/RNS) in different types of CNS immune cells were evaluated *via* the Cellular ROS/RNS Detection Assay Kit (Abcam; catalog number: ab139473) according to the manufacturer’s instructions. Briefly, brain single-cell suspensions were prepared *via* a Percoll density gradient and then treated with a mixture of two different fluorescent dye reagents in phenol-free culture media, allowing the detection of ROS and RNS. After the ROS/RNS dye treatment, the dyed cells were washed, and Fc receptors were blocked with anti-mouse CD16/32 (BioLegend). These cells were then stained with fluorochrome-conjugated antibodies (anti-mouse-BV421-CD45) and anti-mouse-PE-O4 (Miltenyi Biotec) for 40 min at 4 °C to characterize oligodendrocytes (CD45^-^O4^+^). The cells were washed twice and resuspended in FACS staining buffer. The fluorescently stained samples were analyzed on an Attune N x T cytometer (Thermo Fisher Scientific) *via* FlowJo software.

### Bioenergetics

To monitor the mitochondrial oxygen consumption rate (OCR) and extracellular acidification rate (ECAR) in intact cells, a Seahorse Bioanalyzer (Agilent) was used. The XFe mitochondrial stress test was performed on CD4+CD25-cells (purity ∼94%) or F4/80 macrophages (purity ∼92%) isolated from CFA or EAE mice treated with or without MaR1 (300 ng/mouse daily) to measure the OCR. The cells were seeded at a concentration of 0.5 million per well (in the case of CD4+ cells) or 0.1 million per well (in the case of F4/80+) in a polylysine-coated XFe 96-well Seahorse culture microplate in 75 μl of OCR or ECAR DMEM and then centrifuged at 2000 rpm for 1 min. Then, 100 μl of the appropriate medium was added, and the mixture was incubated at 37 °C in a CO_2_-free incubator for degassing. After incubation, the OCR and ECAR were measured according to the manufacturer’s protocol. To measure ATP levels, the cells were plated in 96-well plates (25,000 cells/well), and the ATP concentration was determined with a fluorometric ATP determination kit following the manufacturer’s protocol (Thermo Fisher Scientific). The data are presented as the unit/number of cells.

### Single cell ENergetIc metabolism by profiling translation inHibition

We used the SCENITH assay, a FACS-based, quantitative analysis of mRNA translation in single cells (35, 36, 37, 83). This system takes advantage of cells exposed to a short pulse of PURO, which is incorporated into polypeptides that are being translated, which is an energy-consuming process (84). The fixed cells were permeabilized and stained with an anti-PURO antibody. SCENITH is a proxy for the metabolic fitness of the cell, and its metabolic outcome correlates well with that of the Seahorse Analyzer (35, 36), requiring relatively less number of cells. This approach provides information on the use of energetic pathways (glucose dependence, mitochondrial dependence, glycolytic capacity, and fatty acid and glutaminolysis capacity) (35, 37). This method also allows simultaneous analysis of the metabolic activities of diverse cell populations, such as CD4+ T cells, macrophages, microglia, and O4+ oligodendrocytes, *via* a previously described protocol and optimized assay conditions in our laboratory (37), and the gating strategy is reported in Fig. S1.

### Efferocytosis assay

For the efferocytosis assay, FITC-labeled apoptotic splenic cells devoid of monocytes/macrophages were added to bone marrow–derived macrophages (BMDMs) at a 1:5 ratio for 18 h, followed by five washes with PBS to remove unbound apoptotic cells. The macrophages were then harvested with trypsin and centrifuged, and the pellet was mixed with FACS stain buffer. The macrophages were then stained with anti-mouse BV421-F4/80 for 30 min in the dark at 4 °C. Flow cytometry was used to investigate the engulfment of apoptotic cells (FITC+) by BMDMs. The gating strategy for the efferocytosis assay is reported in Fig. S2.

To demonstrate that BMDMs engulf apoptotic cells, BMDMs were grown on a 4-chamber slide covered with poly-L-lysine. FITC-labeled apoptotic splenic cells were then incubated for the same duration as indicated previously. After eliminating any unbound apoptotic cells, the BMDMs were washed five times with PBS. The samples were then fixed and permeabilized with 4% paraformaldehyde and 0.1% Triton X-100. The BMDMs were then stained with phalloidin and 4′,6-diamidino-2-phenylindole and imaged *via* a confocal microscope.

### RNA sequencing

For transcriptome analysis, the spinal cords of three mice from each group, CFA-, EAE-, and MaR1-treated mice, were subjected to RNA-seq *via* the services of CD-Genomics. The final library size was approximately 400 bp, and the insert size was approximately 250 bp. Illumina 8-nt dual indices were used. Equimolar pooling of the libraries was performed on the basis of the quality check (QC) values, and the libraries were sequenced on an Illumina NovaSeq 6000 (Illumina) with a read length configuration of 150 PE for 40 M PE reads per sample (20 M in each direction). To assess the sequencing quality, a QC was performed *via* FastQC in the data analysis, in which all the samples passed. Using the STAR aligner, paired-end reads of 150 bp were uniquely aligned to GRCm38-mm10 at a mapping rate of approximately 80% STAR to count the reads for each sample. The limma-Voom package in R was used to assess differential expression, with a padj cutoff of 0.05 and a logFC of 0.34. The threshold for filtering out genes with low expression was set to 1 CPM in at least 80% of the samples. The trimmed mean of M values method was used to normalize the counts. Significantly dysregulated genes were analyzed for GO enrichment with an FDR cutoff of <0.05 *via* ShinyGO.

### Peripheral blood cell isolation from MS patients and T-cell responses by flow cytometry

PBMCs were isolated after venous puncture from patients with RR-MS (Table 1) and were separated by density gradient centrifugation over Ficoll-Hystopaque. PBMCs (1 × 10^6^ cells) were pretreated with or without MaR1 (10 nM) for 30 min and then stimulated with Dynabeads CD3/CD28 T-cell Expander (one bead per cell; Invitrogen) for 8 h, as previously reported (46, 85). To measure the intracellular cytokine levels, secretion was inhibited by the addition of 1 μg/ml brefeldin A (Sigma‒Aldrich) 6 h before the end of stimulation with either PMA/ionomycin or Dynabeads CD3/CD28 T-cell expander. At the end of incubation, the cell surfaces were stained with Brilliant Violet 421-conjugated anti-CD3 (BioLegend), FITC-conjugated anti-CD4 (eBioscience), and PerCP5.5-conjugated anti-CD8 (BioLegend) antibodies. The cells were then permeabilized with Cytofix/Cytoperm reagents (BD Biosciences) and stained intracellularly with phycoerythrin-Cy7-conjugated anti-TNF-α (BD Biosciences), allophycocyanin-conjugated anti-IFN-γ (BD Biosciences), and phycoerythrin (PE)-conjugated anti-IL-17 (eBioscience) in 0.5% saponin at RT for 30 min. For Treg staining, the cell surfaces were stained with FITC-conjugated anti-CD4 (BD Biosciences), allophycocyanin-conjugated anti-CD25 (BD Bioscience), fixed and permeabilized with the FoXP3 Transcription Buffer Kit (eBioscience), and intracellularly stained with PE-CF594-conjugated anti-Foxp3 (BD Biosciences) and PE-conjugated anti-IL-10 (BD Biosciences). All samples were acquired on a 13-color CytoFLEX flow cytometer (Beckman Coulter), and for each analysis, at least 300,000 events were acquired by gating on Pacific, Orange-conjugated live/dead negative cells and analyzed by FlowJo Software.

### Data and statistical analysis

All values are presented as the means ± SEMs, and statistically significant differences were assessed *via* the Mann‒Whitney test, Student’s *t* test, and one-way ANOVA. The *p* values for statistical significance are described in the figure legends for each experiment. All the statistical analyses were performed with GraphPad Prism.

## Data availability

All the data generated or analyzed during this study are included in this article and its supplementary information files.

## Supporting information

This article contains supporting Information.

## Conflict of interest

The authors declare that they have no conflicts of interests with the contents of this article.
