# Supplementary material for: Maresin-1 promotes neuroprotection and modulates metabolic and inflammatory responses in disease-associated cell types in preclinical models of multiple sclerosis
Source: J Biol Chem. 2025 Jan 27;301(3):108226. doi: 10.1016/j.jbc.2025.108226 (PMC11903811; doi:10.1016/j.jbc.2025.108226)
Supplement: Supporting information [file mmc1.pdf]

Supporting Information

Maresin-1 promotes neuroprotection and modulates metabolic and inflammatory responses in disease-associated cell types in preclinical models of Multiple Sclerosis

Insha Zahoor[1*], Mohammad Nematullah[1*], Mohammad Ejaz Ahmed[1], Mena Fatma[1], Sajad Mir[1], Kamesh Ayasolla[1], Mirela Cerghet[1], Suresh Palaniyandi[2,3], Veronica Ceci[4,5], Giulia Carrera[5], Fabio Buttari[6,7], Diego Centonze[6,7], Yang Mao-Draayer[8], Ramandeep Rattan[9], Valerio Chiurchiù[4,5], Shailendra Giri[1#]

Supplementary data

Supp Fig 1

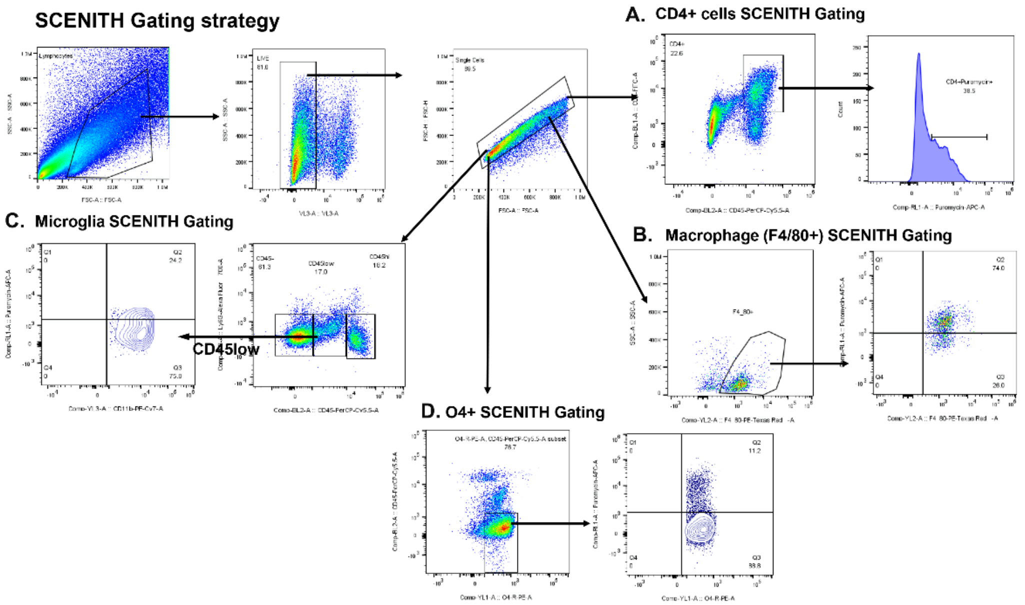

Supp Fig 1: Gating a strategy for immunophenotyping using SCENITH. The brain and spinal cord single-cell suspensions were stained for lymphoid and myeloid markers, as described in the methods section. To eliminate doublets and dead cells, the Live_Dead-ve population was gated, and the FSC-A vs FSC-H data were plotted from negative populations, identifying these populations as live cells, which were used in all subsequent analyses. CD4+ T-cell populations were identified by double gating of CD45+CD4+ cells, and puromycin-positive cells were gated from this population. Microglial populations were gated on CD45lowCD11b+ and puromycin double-positive cells. To detect puromycin+ macrophage populations, CD45+F4/80+ cells were gated for total macrophages, followed by puromycin-positive populations.

Supp Figure 2

***In Vitro* microglia efferocytosis Gating**

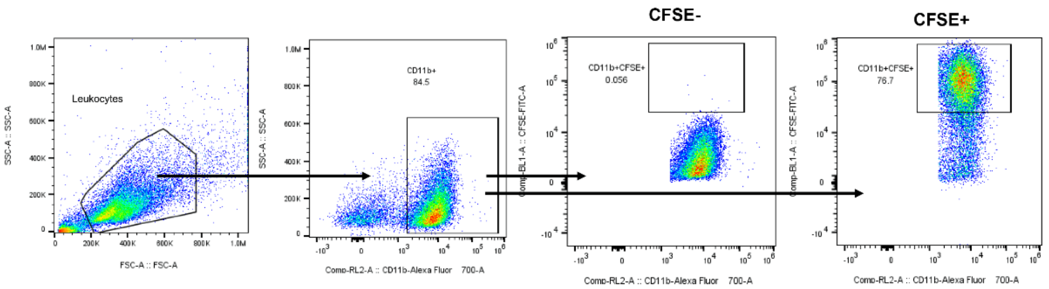

Supp Fig 2: Gating strategy for e*x vivo* efferocytosis analysis. A pseudocolor map was used to quantify total efferocytosis in macrophages and microglia from mice subjected to EAE and treated with MaR1. Double-positive gating of CD11b+CFSE+ populations revealed apoptotic cell engulfment by macrophages and microglia, whereas CD11b+CFSE- populations were identified as nonefferocytotic macrophages and microglia.

Supp Figure 3

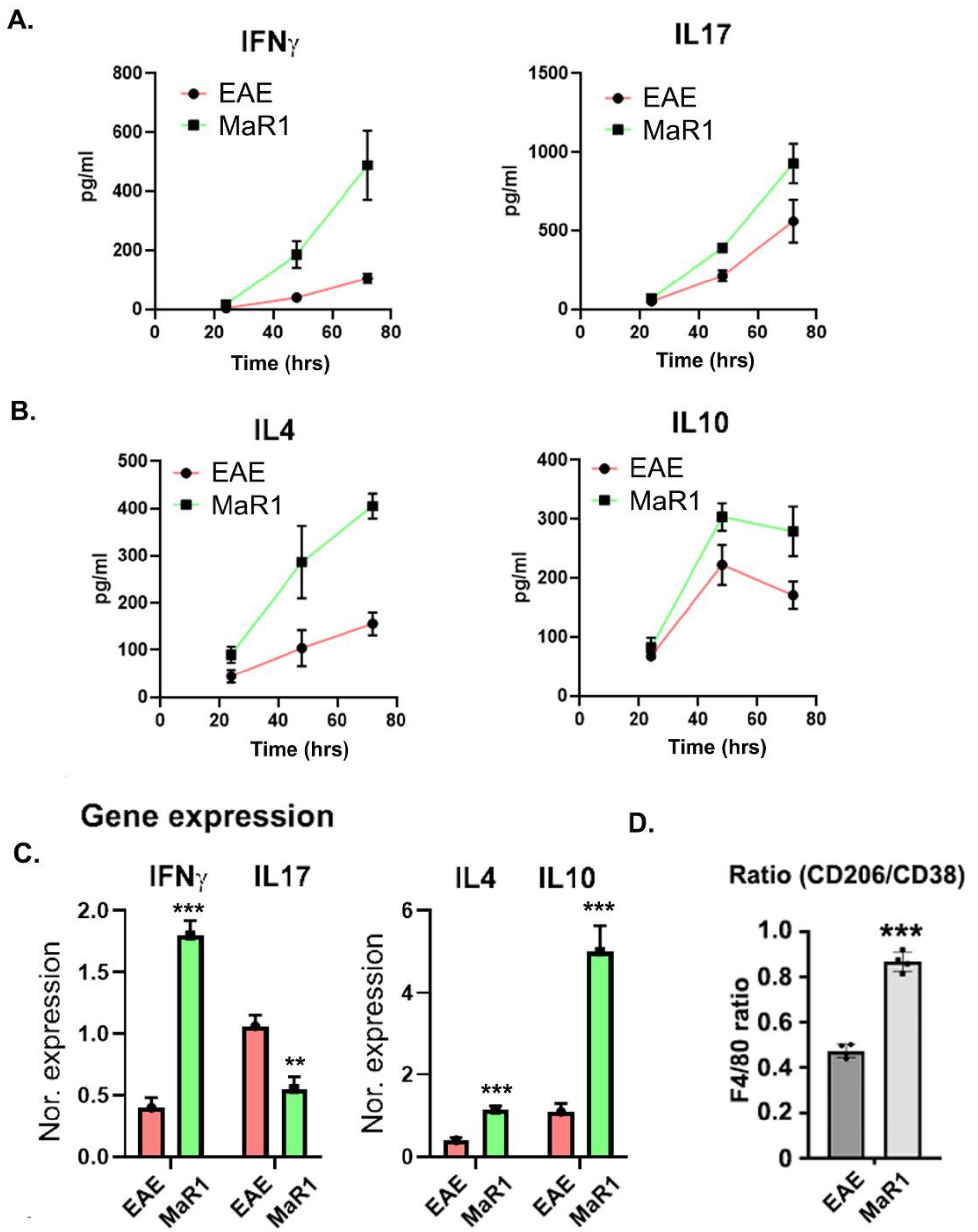

Supp Fig. 3: MaR1 modulated the expression of pro- and anti-inflammatory cytokines in spleen/LN cells and altered the phenotype of macrophages in the CNS. A-B. Time course of antigen-specific production of pro- (IFNγ and IL17a) and anti-inflammatory (IL4 and IL10) cytokines in the spleen/LN cells of the EAE and MaR1-treated groups (n=4). **C.** RNA was isolated from spleen/LN cells from mice with untreated EAE and MaR1 after 24 h

of antigen stimulation, after which the expression of IFNγ, IL17a, IL4 and IL10 was examined (n=3). **D.** CNS tissues (brain and spinal cord together) were processed, and CD206+ and CD38+ F4/80+ cells in the CNS of treated and untreated RR-EAE (n=4) were examined. The ratio of CD206/CD38+ macrophages was plotted to determine the macrophage phenotype (n=4) in response to MaR1 treatment compared with vehicle-treated EAE. **, P<.01; ***, P<.001.

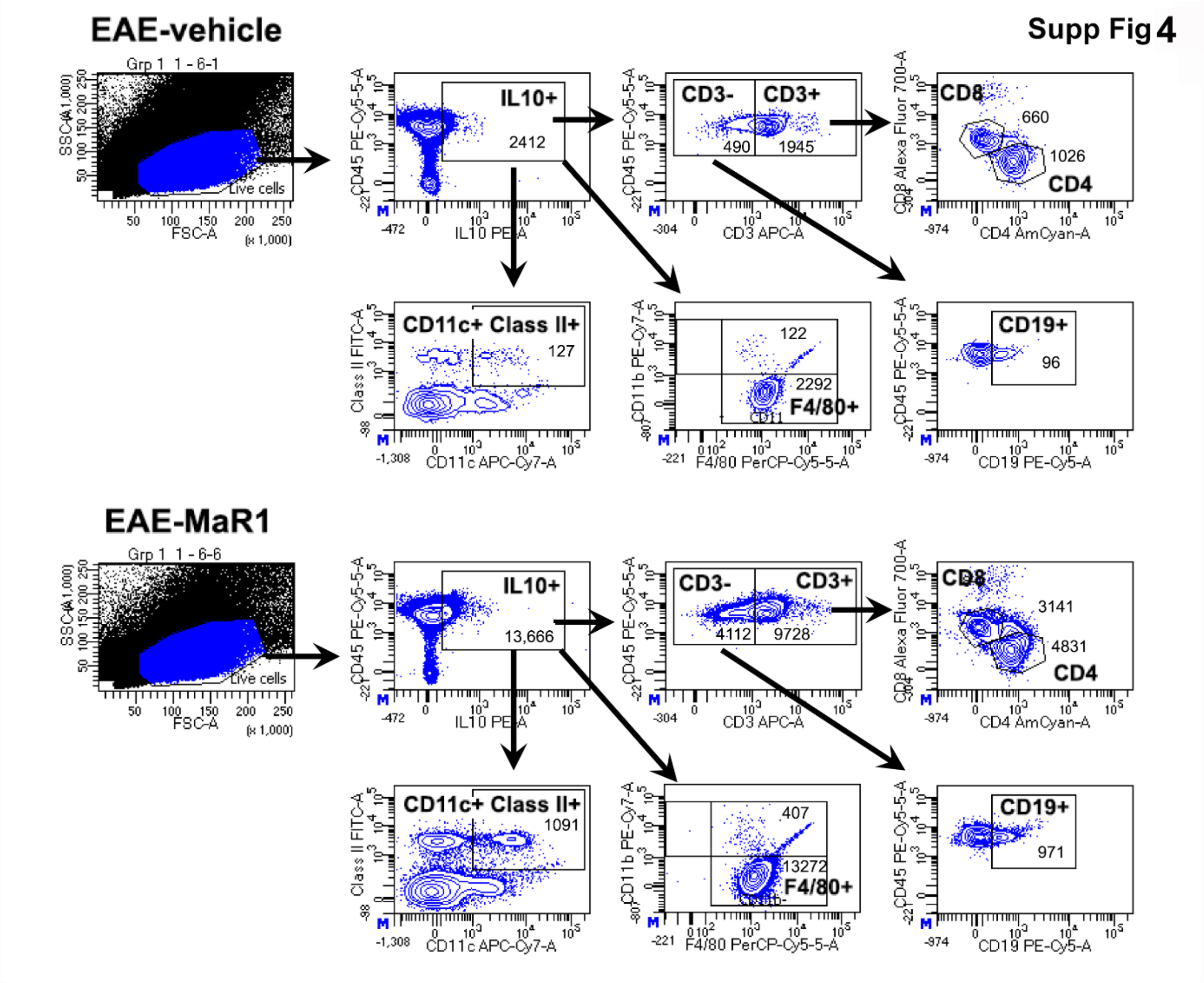

Supp Figure 4: Gating strategy for examining IL10-expressing immune cells. Using a Percoll density gradient, brain and spinal cord single-cell suspensions from EAE and MaR1-treated EAE mice were generated and subjected to flow cytometry analysis. Before identifying IL10-expressing lymphoid cells, CD3+ populations were gated from IL10+ populations. CD4+ and CD8+ T cells were identified in CD3+ populations, whereas B cells were identified in CD3- populations. To identify IL10-expressing myeloid populations, CD11c+Class II+ populations were gated for inflammatory dendritic cells, whereas CD11b+ F4/80+ populations were gated for macrophages from IL10+ populations.

Supp Figure 5

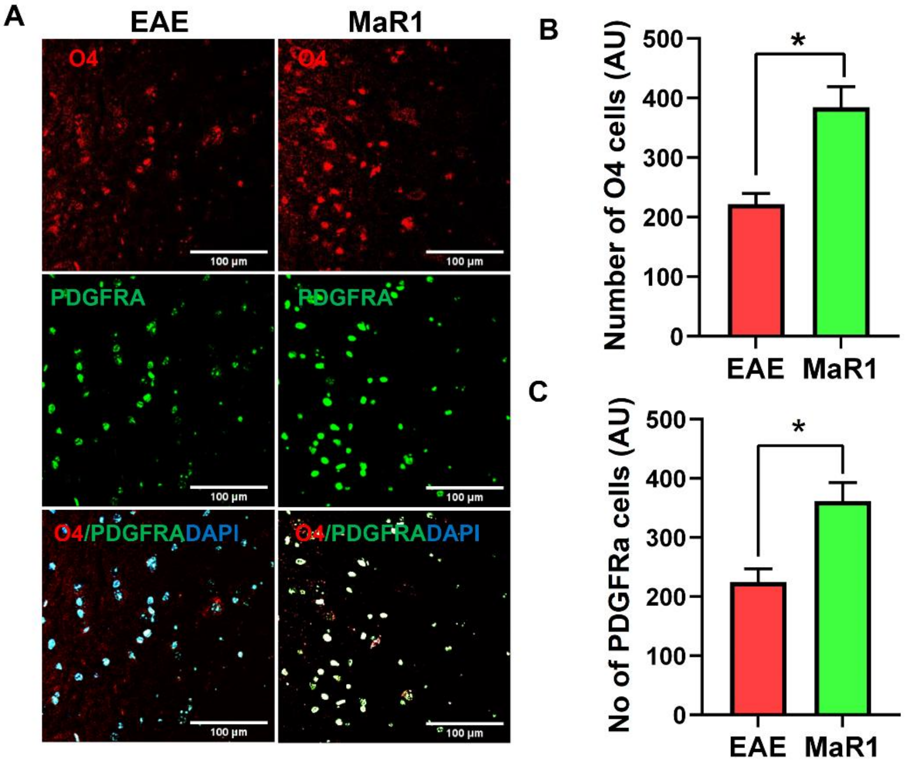

Supp Fig. 5: Effects of MaR1 treatment on oligodendrocyte progenitor cells (OPCs) and premyelinating oligodendrocytes (Pre-OLs) in RR-EAE. Lumbar spinal cord sections were stained with an O4 antibody (red) and PDGFRA antibody (green). **A.** Representative immunofluorescence staining for O4 (a marker for pre-OLs) and PDGFRA (a marker for OPCs) was performed on lumbar spinal cord sections from EAE mice treated with or without MaR1. Scale bar = 100 µm. **B-C.** Quantitative analysis of O4- and PDGFRA-immunoreactive cells. The data are presented as the means ± SEMs (n=5). *P<0.05 vs. EAE mice.
